# Sex-associated and disease state-dependent monocyte polarization and CNS-trafficking phenotypes in pediatric acute-onset neuropsychiatric syndrome (PANS)

**DOI:** 10.1186/s12974-025-03549-6

**Published:** 2025-11-18

**Authors:** Shamma S. Rahman, Noor Hussein, Silvia Giulia Galfrè, Fabian Gaertner, Claudia Macaubas, Avis Chan, Laurie Columbo, Jaynelle Gao, Samira Galehdari, Batuhan Bayram, Meiqian Ma, Cindy Manko, Kate Miles, Bahare Farhadian, Melissa Silverman, Margo Thienemann, Noga Or-Geva, Keith Van Haren, Kari C. Nadeau, Lu Tian, Jennifer Frankovich, Elizabeth D. Mellins

**Affiliations:** 1 Department of Pediatrics, Division of Human Gene Therapy, Program in Immunology, Stanford University School of Medicine, Stanford, USA; 2 Department of Computer Science, University of Pisa, Pisa, Italy; 3 Department of Pediatrics, Division of Allergy, Immunology Rheumatology, Stanford University School of Medicine, Stanford, USA; 4 Stanford Immune Behavioral Health Clinic and PANS Research Program at Lucile Packard Children’s Hospital, Stanford, USA; 5 Department of Psychiatry and Behavioral Sciences, Division of Child and Adolescent Psychiatry and Child Development, Stanford University School of Medicine, Stanford, USA; 6 Department of Neurology and Neurological Sciences, Stanford University School of Medicine, Stanford, USA; 7 Department of Biomedical Data Science, Stanford University School of Medicine, Stanford, USA

## Abstract

Pediatric acute-onset neuropsychiatric syndrome (PANS) is characterized by the sudden onset of obsessive-compulsive symptoms alongside a constellation of neuropsychiatric and somatic features. Disease progression typically includes flare and recovery states, with some patients exhibiting a persistent disease course (> 12 months of flare). We characterized circulating monocyte subsets during flare and recovery in pediatric patients with PANS, uncovering disease-state–dependent shifts in polarization and trafficking phenotypes. Inflammatory M1-like monocytes and monocyte-derived dendritic cells were elevated during flare, while anti-inflammatory M2-like monocytes were enriched in recovery. We also identified a circulating subset with a surface phenotype consistent with central nervous system (CNS) homing, which was reduced during flare and restored in recovery. These cells were detectable in the cerebrospinal fluid (CSF) of new-onset patients but not in persistent cases, suggesting differential compartmentalization during disease progression. Notably, monocyte phenotypes, including M2 polarization (monocytosis) and circulating CNS-homing profiles, exhibited striking sex-associated differences, particularly during recovery, with several subsets enriched in males but not females. Plasma from flare-phase patients modestly induced CNS-homing markers in monocytes from healthy donors, indicating the presence of circulating modulators. Together, these findings support a model in which distinct myeloid phenotypes—including sex-biased patterns—may contribute to both the pathogenesis and resolution of neuroinflammation in PANS.

**Supplementary Information:**

The online version contains supplementary material available at 10.1186/s12974-025-03549-6.

## One-sentence summary

Circulating monocyte phenotypes in pediatric PANS vary by disease state and sex, revealing immune polarization and CNS-trafficking profiles that may underlie neuroimmune symptom dynamics.

## Significance statement

PANS is a debilitating neuroimmune condition of unknown etiology that imposes significant morbidity on affected children and a high caregiving burden on families. Our findings support a model in which distinct sex-associated myeloid cell subsets contribute to both the pathogenesis and resolution of PANS flare. We identified a novel subset of CD14⁺ monocytes with a regulatory, anti-inflammatory profile and a surface phenotype consistent with CNS homing. These “CNS-homing” monocytes were elevated in the blood during recovery and detected in the CSF during flare, suggesting active redistribution across neuroimmune barriers. To our knowledge, this is the first report of such cells in any human population. Given their disease-state- and sex-associated expression and potential regulatory function, these monocytes or their molecular signatures may serve as biomarkers of disease trajectory, aid in clinical decision-making, and offer new therapeutic entry points in PANS.

## Introduction

Pediatric acute-onset neuropsychiatric syndrome (PANS) is a neuropsychiatric disorder, characterized by abrupt-onset and a relapsing-remitting course [1–4]. Relapses (flares) are characterized by new-onset or dramatic escalation in obsessive-compulsive symptoms and/or eating restriction, in addition to escalation in other neuropsychiatric symptoms: anxiety, emotional lability, mood dysregulation, strong irritability and/or rage, behavioral regression, motor abnormalities (e.g. tics or choreiform movements), inattention, learning difficulties, sleep dysregulation, sensory dysregulation, and urinary frequency and/or enuresis [1, 5–8]. Typical onset age is 5–9 years, and male predominance has been observed [2, 9–11]. While the vast majority of patients with PANS are on a relapsing-remitting course, many patients also have periods of persistent disease (flare state is ongoing for > 12 months without a period of an unequivocal recovery and flares-on-persistent) [4].

Sex is an increasingly recognized biological variable in neuroimmune and neurodevelopmental disorders. Males and females exhibit distinct immune responses influenced by sex hormones and sex chromosome-linked regulation, which can affect both innate and adaptive immunity [12]. In PANS, early clinical studies and cohort characterizations have consistently reported a Male-to-female ratio of approximately 3:2 [2–4, 9, 11, 13]. and different clinical features between sexes. For example, one study found that males exhibited more aggressive behavior than females [14]. Another study found that post-pubertal females had higher rates of persistent disease and emotional lability, suggesting sex-related variation in disease chronicity and symptom expression [15]. Despite these observations, the biological underpinnings of sex differences in PANS remain poorly understood, and the role of immune cell phenotypes in mediating sex-associated disease trajectories has not been systematically explored.

PANS is thought to arise from a post-infectious inflammatory condition which may be both systemic [16] and involve the basal ganglia (a key region of the brain which governs mood, emotion, behavior, procedural learning, movements and is involved in obsessive and compulsive symptoms). Evidence for basal ganglia involvement include: (1) Imaging studies indicating that the most prominent findings are within the basal ganglia including microglia activation, swelling in the acute stage (but not the persistent phase), and microstructural changes [17–20]; (2) Motor disinhibition during rapid eye movement (REM) sleep [21–24], a finding that, in adults, strongly predicts the development of Parkinson’s disease; (3) Neurological soft-signs which reflect basal ganglia involvement, including a positive glabellar tap which is also a predictor of Parkinson’s disease [25] and (4) Autoantibodies which target cholinergic interneurons within the basal ganglia [26–28] and other structures [29–36].

Although the post-infectious nature of PANS [2, 9–11] and detection of autoantibodies which target neurons in active PANS plasma [26–33] argue for involvement of the immune system in pathogenesis, little is known about the roles of specific circulating immune cells in patients with PANS. In most inflammatory/autoimmune conditions, multiple arms of the immune system are involved. In a previous report, we showed increased circulating monocytes in patients with PANS [2]. Interestingly, recent studies of pediatric (early onset) obsessive-compulsive disorder (OCD) patients, who share OCD behavior with patients with PANS, revealed increased circulating pro-inflammatory monocytes [37, 38]. Lastly, a recent study from our group indicates a reduction in pro-inflammatory monocytes in patients with PANS after treatment with IVIG [39]. Thus, monocytes are a candidate cell type to contribute to the physiology of PANS.

Monocytes constitute ~ 2–8% of total blood leukocytes [40]. They express human leukocyte antigen (HLA)-DR, and they contribute to host defense and tissue repair/remodeling. They are highly responsive to environmental cues, including the cytokine and metabolic milieu, and circulating monocytes can reflect inflammatory or resolving processes in tissue [41]. Human blood monocytes have been classified in 3 major subsets, based on their expression of the surface markers CD14 and CD16: classical (CD14 + CD16-, pro-inflammatory), intermediate (CD14 + CD16+, pro-inflammatory or tolerogenic), non-classical (CD14-CD16+, anti-inflammatory), with classical monocytes comprising ~ 80–85% of total circulating monocytes, intermediate monocytes 2–8% of total, and non-classical monocytes 2–15% of total. These subsets are developmentally related, with a small percentage of circulating classical monocytes differentiating sequentially into intermediates and non-classical [42]. Analysis of monocyte subsets continue to evolve, from phenotype to gene expression, and further heterogeneity within the subsets may be present. The three monocyte subsets appear to have distinct biological functions [43].

Furthermore, it has been shown that monocyte-derived macrophages can be functionally polarized towards pro-inflammatory or anti-inflammatory/regulatory states in vitro [44], namely “M1” (pro-inflammatory) and “M2” (anti-inflammatory). Although these activation states likely represent the extremes range of macrophage polarization, they provide a useful framework to analyze the functional phenotype of macrophages and monocytes [44]. Considering the relapsing-remitting nature of PANS in most patients, we hypothesized that both pro- and anti-inflammatory monocyte subsets could be observed in association with distinct PANS clinical states.

Given the reported findings of basal ganglia changes in patients with PANS, we also hypothesized that a subset of monocytes might home to the central nervous system (CNS) during PANS flare. Based on previous studies, we chose the following markers as a candidate panel to identify potential “CNS-homing” monocytes: HLA-DR, CD14, CCR2, CX3CR1, VLA-4 and CD166. CCR2, a chemokine receptor for CCL2, is expressed most abundantly on classical monocytes, regulates trafficking of these cells into sites of inflammation [45, 46]. The CX3CR1 chemokine receptor, expressed most abundantly on non-classical monocytes, is required for the adhesion of monocytes to endothelium expressing its ligand, fractalkine (CX3CL1) [47, 48]. Interestingly, in a murine model of repeated social defeat, myeloid cell recruitment into the central nervous system required both CCR2 and CX3CR1 [49]. CCR2 signaling results in a conformational Change in the very late antigen 4 (VLA-4) adhesion molecule on monocytes and increases its binding to vascular cell adhesion molecule 1 (VCAM-1) on endothelium [48]. This change enhances monocyte extravasation and homing to inflammatory sites. In several in vitro and murine models of neuroinflammatory disease, VLA-4/VCAM interaction is key to monocyte crossing of the blood brain barrier (BBB) [50–52]. In addition, CD166 (ALCAM) on CD14^+^ monocytes is involved in their rolling, adhesion and diapedesis across the BBB through homotypic interaction with CD166 on brain endothelial cells [53–55]. In this study, we analyzed the phenotype of monocytes and other myeloid cells from patients with PANS during different disease states and clinical courses: flare vs. recovery (partial or full) and relapsing-remitting (RR) course vs. persistent course.

## Materials and methods

### Samples

This study protocol was approved by the Stanford Human Participants institutional review board and covers all patients in this study. Immune Behavioral Health (IBH) Clinic clinicians cared for all patients. Written parental consent and child assent were obtained per study protocol (protocol number 26922). All study subjects were classified as PANS by the IBH Psychiatry team using PANS classification and evaluation criteria [5]. Disease state and clinical course definitions are shown in Supplementary Table 1 [4]. Psychiatric symptoms at clinic presentation and other demographics for PANS samples used in each experiment are listed in Supplementary Tables 2–4. Paired PANS flare and recovery samples were used whenever possible. PANS subgroups (see Supplementary Table 1 for definition) included in this study were: new-onset flare (early in their flare and disease course) *n* = 6 Males, 6 females (with paired recovery in all the Males and 2 of the females); patients on a persistent PANS course (> 12 months of continuous flare symptoms with no period of recovery at the time of specimen collection), *n* = 8 (4 Males, 4 females with paired recovery in 3 Males and 2 females); flare-on-persistent course, *n* = 16 (8 Males, and 8 females with paired recovery in all 16). At the time of blood draw, 49% of patients were receiving NSAIDs, 14% were on hydroxychloroquine, and 5% were on mycophenolate mofetil. Importantly, no patients had received intravenous immunoglobulin (IVIG) within 3 months of sampling, and only 2 patients (5%) had been exposed to corticosteroids within 4 weeks of blood draw. Six patients (16%) were non-medicated at the time of sampling. Paired flare-recovery samples were prioritized where available to minimize intra-individual confounding by treatment exposure. Samples from non-PANS-related disorders included samples from patients with asthma (*n* = 6) and non-PANS-related brain disorders (adrenoleukodystrophy, *n* = 3; temporal lobe epilepsy, *n* = 1; voltage-gated potassium channel antibody syndrome, *n* = 1). For each experiment, PANS samples were matched (to the best of our ability) with samples from healthy controls based on age (+/- 1 year), sex, and race/ethnicity. Blood samples were processed using Ficoll gradient to acquire peripheral blood mononuclear cells (PBMC) and plasma; these were stored in liquid nitrogen and − 80°C, respectively, and maintained by Stanford Biobank.

### Multiparameter flow cytometry

High dimensional flow cytometry was performed according to our established protocols, with modifications [56]. Briefly, PBMC were thawed in a 37°C water bath and spun at 400 g at 4°C for 5 min. The supernatant was discarded, and the pellet was resuspended in blocking buffer (5% Heat inactivated AB human sera, 5% normal goat serum in PBS) at the desired concentration (2 × 10^6^ cells/50 µl). Samples were distributed in a 96- deep-well v-bottom plate (50 µl/well) and the cells were incubated for 15 min on ice. After the 15-min incubation, the prediluted antibodies were added directly to the cells and mixed gently by pipetting three times. Cells were allowed to incubate for 30 min on ice and in the dark, followed by wash with FACS Buffer (1x PBS, 0.5% bovine serum albumin - BSA, 0.02% sodium azide). Then cells were stained with live/dead^™^ dye Aqua (ThermoFisher Scientific) prior to fixation and permeabilization using BD Cytofix/Cytoperm™ kit (BD Biosciences) and then subjected to staining with intracellular antibodies. The compensation controls were done with UltraComp eBeads (ThermoFisher Scientific) for all antibodies. One drop of the bead solution was mixed with 100 µl of FACS Buffer and 5 ul of antibody. The compensation control for the live/dead dye Aqua was generated using the ArCTM Amine Reactive Compensation Bead Kit (ThermoFisher Scientific). One drop of the bead solution was mixed with 1 µl of the Aqua dye and 100 ul PBS. The tubes were incubated in the dark on ice for 15 min, followed by resuspension in FACS Buffer (or PBS). The tubes were spun at 400 g for 10 min. The supernatant was discarded and 200 µl FACS buffer was added. The compensation controls were kept at 4°C until the acquisition. After the final washes, cells were resuspended in 200 ul FACS buffer and data was acquired using a LSRII.UV machine available at the Stanford Shared FACS Facility. The cell count was performed using BD Trucount™ (BD Biosciences) as directed by the manufacturer.

### Antibody panels

Unless specified, all antibodies were purchased from BioLegend and were used at the concentration specified by the Manufacturer. The antibodies used to identify monocytes, dendritic cells, Macrophage-like cells in peripheral blood mononuclear cells are listed in Supplementary Tables 5, 6, 7 and 8.

### CSF cell staining

CSF samples were collected from patients with PANS during acute flare as part of their initial clinical diagnostic workup at the Stanford PANS Clinic. All lumbar punctures were performed prior to the initiation of immunomodulatory therapy. Freshly obtained CSF was processed immediately. The total number of viable cells was determined using trypan blue exclusion. Cells were then pelleted by centrifugation, resuspended in staining buffer, and stained with fluorophore-conjugated antibodies for flow cytometry analysis.

### Intracellular cytokine flow cytometry

Whole blood samples collected from patients with PANS and age/sex/race/ethnicity-matched healthy controls were immediately fixed using proteomic stabilizer (SmartTube Inc.) and stored in −80°C until ready for use. Fixed whole blood was sequentially thawed, first at 15°C for 20 min, and then at room temperature for 10 min. Red blood cells from the thawed samples were lysed using Thaw-Lyse buffer (SmartTube Inc.) at a concentration of 1:1000. Cells were incubated in 25 mL Thaw-Lyse buffer for 10 min, followed by centrifugation at 400 g for 10 min at room temperature. After two rounds of lysis, cells were visually clear of all red blood cells, and the intracellular production of anti-inflammatory and proinflammatory cytokines was measured. The cells were stained for extracellular dump channel markers (CD235a, CD15,CD3,CD19,CD56,CD66b) to exclude erythrocytes, granulocytes T cells, B cells, NK cells, and neutrophils, respectively from the analysis. The cells were also stained with “CNS-homing” monocyte surface markers panel (HLA-DR, CD14, CCR2, CX3CR1, VLA-4, CD166), then permeabilized using BD Cytofix/Cytoperm™ kit (BD Biosciences), and stained for intracellular IL-10, TGF-β, GM-CSF, IL-6, and TNF-α (see Supplementary Table 8 for flow cytometry antibody characteristics and Supplementary Table 9 for the cytokines functions). The compensation controls were done with UltraComp eBeads (ThermoFisher Scientific) for all antibodies as above. FACS analysis was carried using FACSymphony^™^ A5 celL analyzer (BD Biosciences) at the Stanford Shared FACS Facility.

#### Single- cell RNA sequencing (scRNA-seq)

PBMCs samples were collected from four male patients with PANS (in flare and in recovery), pelleted, viability determined using trypan blue exclusion, and resuspended in 0.04% bovine serum albumin (BSA) in PBS. Samples were loaded onto 10X Genomics Chromium platform for Gel Beads-in-emulsion (GEMs) and cDNA generation carrying cell- and transcript-specific 10X barcodes. Subsequently, single cell RNA sequencing libraries were constructed using Chromium Single Cell 3′ Library and Gel Bead Kit v3 as previously described [57]. The generated libraries were sequenced on the Illumina NovatSeq6000(Novogene) using pair-ended sequencing, targeting a depth of 100,000 reads per cell.

#### scRNA-seq data processing and analysis

##### Single-Cell data preprocessing

PBMCs samples from four different patients with PANS (with F indicating flare and R for recovery) and three different healthy control FASTQ data for single-cell RNA sequencing from 10x Genomics support site were analyzed. FASTQ reads were aligned, filtered, and counted through the Cell Ranger pipeline (v4.0) provided by 10x Genomics using standard parameters, with alignment performed against the GRCh38 (hg38) human reference genome. After the initial cleaning using Droplet Utils to remove the empty droplets [58, 59], COTAN [60] was used to performs an initial clustering and drops low quality clusters. Doublet Finde r [61] was used to remove doublets.

##### Dimensionality reduction and clustering

After the single-cell data pre-processing, the raw Matrices were merged, and the cells were automatically identified using Azimuth 0.5.0. Cells corresponding to the CD14 Mono class (predicted.celltype.l2) were retained (verification performed twice). Next, standard Seurat v5 dataset integration was conducted using 25 PCA dimensions and the CCAIntegration method (considering each different patient a batch), as previously described [62, 63]. To better distinguish the “CNS-homing” from the “non-CNS-homing” monocytes, we applied a selection approach like gating. We cluster the cells (FindClusters with resolution of 2) removal of CD16 + cells identified using FindAllMarkersGenes and removing clusters significantly enriched for the CD16 gene (adjusted p-value < 0.05). Exclusion of CCR2-negative clusters using FindAllMarkersGenes and dropping the ones depleted in CCR2 (avg_log2FC < 0 with adj. p-value < 0.05). Selection of the “non-CNS-homing”cells: applied FindAllMarkersGenes, retaining only clusters depleted in CX3CR1 (avg_log2FC < 0 with adj. p-value < 0.05 – repeated the check twice). Final refinement of the “CNS-homing”: after removing the “non-CNS-homing” cells identified in the previous step, the remaining clusters were enriched in VLA4. We further refined the selection by removing clusters depleted in CD166 and any clusters with fewer than 50 cells (to reduce noise). The remaining cells were classified as “brain -homing” monocytes. This procedure resulted in 2,025 “non-CNS-homing” cells and 5,255 “CNS-homing” cells, distributed as shown in Supplementary Table 10.

##### Differential expression and pathway enrichment analyses

Differential expression analysis between the “CNS-homing” and “non-CNS-homing” cells was performed using Seurat FindAllMarkersGenes function. Over representation analysis on the up-regulated and down-regulated genes was then carried out using WebgestaltR tool (version 1.0.0) applying as background genes the expressed genes (count greater than 0) using AggregateExpression function from Seurat.

### Statistics

Data acquired from flow cytometry experiments were analyzed by FlowJo v.10 software (FlowJo LLC), and cells were identified using a manual gating strategy, shown in Supplementary Figure S1. All data were analyzed using GraphPad Prism v8. Overall comparisons between > 2 groups were done with Kruskall–Wallis tests; pair-wise comparisons were performed by Mann–Whitney tests. Comparisons of paired samples were done using the Friedman test to account for within pair correlations. For all tests, a P value less than 0.05 was considered statistically significant.

## Results

### Monocytosis is observed in male patients with PANS during flare

We first compared total monocyte counts in unstimulated peripheral blood of patients with PANS to healthy controls using flow cytometry (gating strategy on Supplementary Fig. 1A). We analyzed male and female cohorts separately, as peripheral monocyte counts in male healthy subjects were higher than those in female healthy subjects (Tables 1 and 2), consistent with previous reports [64].

**Table 1:** Monocytosis in male patients with PANS in flare reflects increased Circulating classical monocytes

Cell counts (median ± SEM)	Male			*P* value		
	Healthy control (*n* = 10)	PANS flare (*n* = 17)	PANS recovery (*n* = 17)	HC* vs. flare	flare vs. recovery	HC* vs. recovery
Total monocytes (HLA-DR^+^CD14^+^CD16^+^)	396,690 ± 236,759	780,538 ± 498,567	619,649 ± 674,930	0.002	0.34	0.18
Classical monocytes (HLA-DR^+^ CD14^+^CD16^−^)	321,055 ± 57,903	627,440 ± 90,289	390,591 ± 78,119	0.016	0.18	0.73
Non-classical monocytes (HLA-DR^+^CD14^−^CD16^+^)	91,226 ± 11,980	124,066 ± 9320	100,024 ± 7230	> 0.999	> 0.999	> 0.999
Intermediate monocytes (HLA-DR^+^CD14^+^CD16^+^)	6405 ± 892	2747 ± 321	4989 ± 927	0.006	0.37	0.25

*Healthy controls (HC): age/sex/race/ethnicity matched to PANS cases

**Table 2:** Absence of monocytosis in female patients with PANS

Cell counts (median ± SEM)	Female			*P* value		
	Healthy control* (*n* = 10)	PANS flare (*n* = 17)	PANS recovery (*n* = 17)	HC* vs. flare	flare vs. recovery	HC* vs. recovery
Total monocytes (HLA-DR^+^ CD14^+^CD16^+^)	393656.4 ± 372,273	396262.3 ± 615,209	298389.2 ± 511,192	> 0.999	> 0.999	> 0.999
Classical monocytes (HLA-DR^+^CD14^+^CD16^−^)	227946.7 ± 392,300	243883.6 ± 287,450	171292.7 ± 169,834	> 0.999	> 0.999	> 0.999
Non-classical monocytes (HLA-DR^+^CD14^−^CD16^+^)	130367.3 ± 70,567	145870.6 ± 225,752	67294.3 ± 324,675	> 0.999	> 0.999	> 0.999
Intermediate monocytes (HLA-DR^+^CD14^+^CD16^+^)	8110 ± 10,930	17,682 ± 58,942	6311 ± 52,410	0.48	> 0.999	0.52

*Healthy controls (HC): age/sex/race/ethnicity matched to PANS cases

**Fig. 1:**
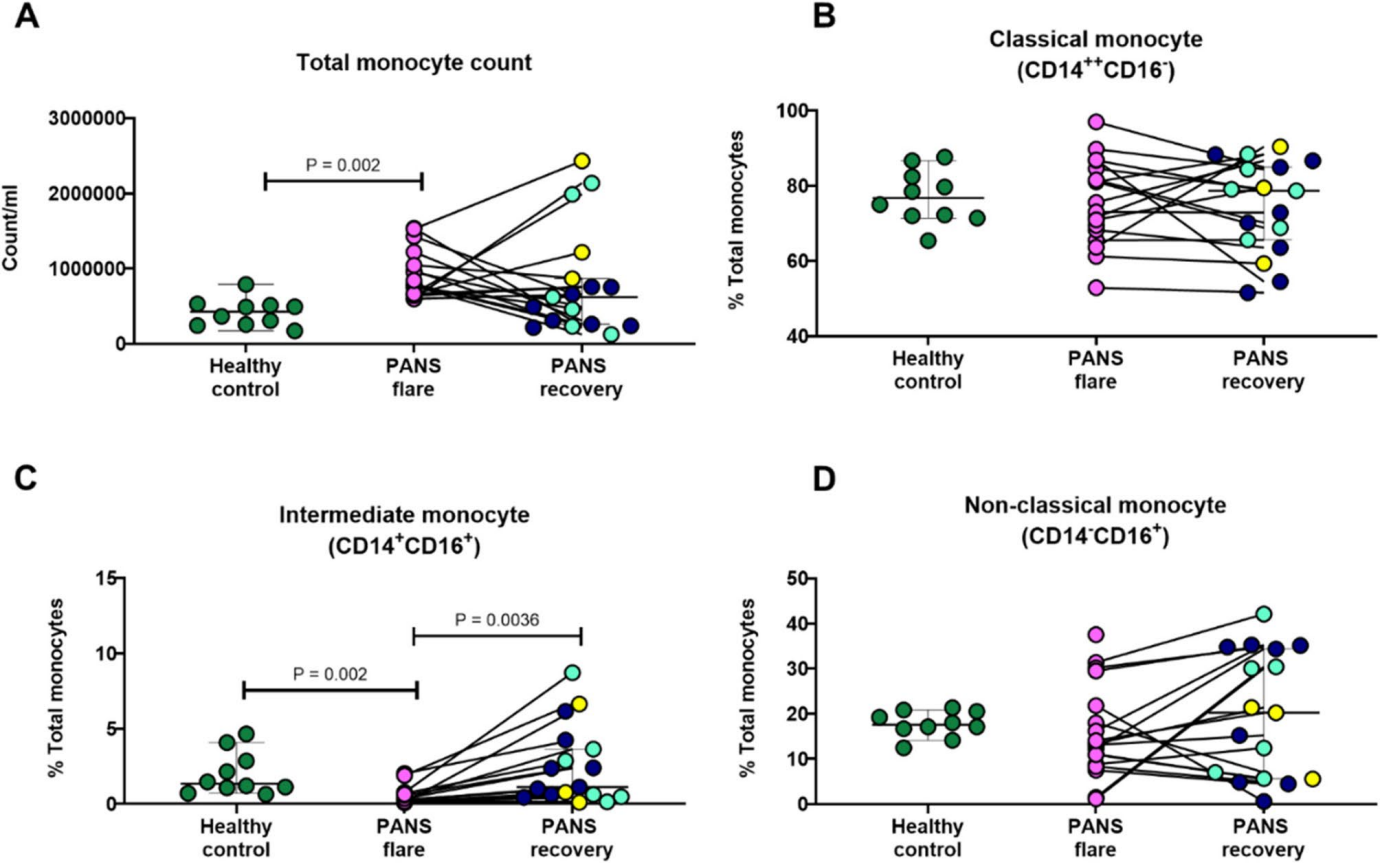
Monocytosis observed in male patients with PANS in flare. Unstimulated PBMCs from male patients with PANS in flare (*n* = 17) and in recovery (partial or full) (*n* = 17) (paired samples, *n* = 17) and age/sex (male)/race/ethnicity-matched healthy controls (*n* = 10), were stained with markers for monocyte subsets and analyzed by flow cytometry. **A** Total monocyte count in male subjects. Proportion (represented as %) of **B** classical, **C** intermediate, and **D** non-classical monocytes.HLA-DR^+^ population was manually gated to identify total monocytes and the three major monocyte subsets: classical (CD14^+^), non-classical (CD16^+^) and intermediate (CD14^+^CD16^+^). CD14^+^ cells were further gated to identify monocyte-derived dendritic cell (mo-DC; see Supplementary Fig. 1). Paired samples are indicated by a straight line and were analyzed by Friedman Test (*P* value < 0.05 indicating significance). Unpaired samples were analyzed by non-parametric Mann-Whitney test (*P* value < 0.05 indicating significance). The different colors displayed in the recovery group represent three sub-groups of patients whose state and course (prior to their recovery) were characterized by: new-onset (light blue), flare-on-persistent baseline (dark blue) and static-persistent (yellow)

The total monocyte number in male patients with PANS in flare was increased compared to age/ethnicity-matched healthy male controls and often fell with disease recovery (partial or full recovery) (Fig. 1A). Only a fraction of female patients with PANS in flare had elevated monocyte counts compared to healthy female controls (age/ethnicity-matched) (Supplementary Fig. 2A) and the group difference did not reach statistical significance. In relation to the three monocyte subsets, we found that the number of classical CD14^+^ monocytes was significantly higher in male patients with PANS in flare compared to healthy controls (Table 1), while the number of intermediate monocytes were decreased in PANS flare compared to healthy controls (Table 1). No differences were observed for non-classical monocytes.

Although the numbers of classical monocytes were increased in male patients with PANS in flare, the percentage of this subset was not significantly different among the groups (Fig. 1B). In male patients with PANS in flare, the proportion of the intermediate monocyte subset (CD14^+^CD16^+^) was significantly lower, consistent with their reduced cell counts, when compared to both healthy controls and samples from individuals with PANS in recovery, as illustrated in Fig. 1C. Notably, the counts of this subset in most male patients with PANS increased upon recovery, returning to counts comparable to those observed in healthy controls, as shown in (Fig. 1C). Meanwhile, no significant changes were noted in the total count of non-classical (CD14^-^CD16+) monocytes, as indicated in (Fig. 1D).

In contrast, in female patients with PANS, although the number of the three monocyte subsets were not different among the groups, the proportion of intermediate monocytes was elevated in PANS flare compared to PANS recovery or healthy controls (Table 2, Supplementary Fig. 2C). No changes were observed for the classical and non-classical subsets for female subjects. In sum, monocyte flow cytometry analyses revealed elevated total monocyte numbers, likely due to an increase in classical monocytes, and a reduction in the number and in the proportion of intermediate monocytes as features of PANS flare in male subjects. These findings were not shared by most female subjects, consistent with sex-linked differences in monocyte behavior.

### M1-polarized and M2-polarized monocytes are increased in PANS flare and recovery (partial or full), respectively

**Fig. 2:**
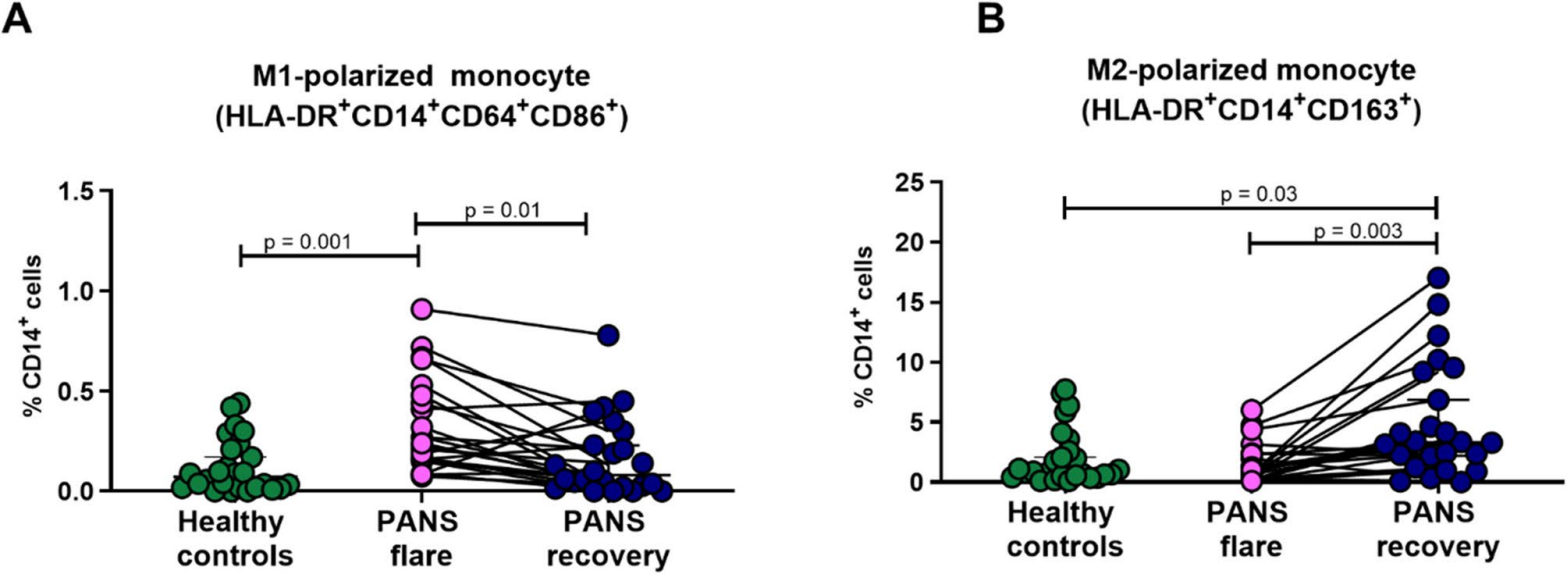
M1-polarized and M2-polarized monocytes are increased in PANS flare and recovery (partial or full), respectively. Unstimulated PBMCs from patients with PANS in flare (*n* = 25) and in recovery (partial or full) (*n* = 25) (paired samples, *n* = 25) and age/sex/race/ethnicity-matched healthy controls (*n* = 25), were stained with markers for polarized macrophage-like cell subsets. These subsets were identified within the HLA-DR^+^CD14^+^ population. CD64 and CD86 were used to identify M1-polarized monocytes (pro-inflammatory). CD163 was used to identify M2-type (anti-inflammatory). **A** Proportion of M1-polarized monocytes in the circulation of patients with PANS, represented as a percentage of total CD14^+^ cells. **B** Proportion of M2-polarized monocytes in patients with PANS, represented as % CD14^+^ cells. Paired samples are indicated by a straight line and were analyzed by Friedman Test (*P* < 0.05 indicating significance). Unpaired samples were analyzed by non-parametric Mann-Whitney test (*P* < 0.05 indicating significance. The state and course (prior to their recovery) were characterized by flare-on-persistent course (dark blue)

To investigate the functional state of monocytes during PANS flare and recovery, we first used the paradigm of monocyte polarization. Monocytes can adopt changes in their surface markers and be polarized toward M1 and M2 states (Supplementary Table 11), as originally observed for macrophages [48, 65]. Polarization toward an M1 or M2 phenotype reflects the inflammatory state of a monocyte, with M1 being typically pro-inflammatory and M2 associated with anti-inflammatory functions. We analyzed the circulating mono/macrophage-like population in unstimulated PBMC from patients with PANS, compared to age/sex/race/ethnicity-matched healthy controls. We observed an increase in the proportion of M1 (CD14^+^CD64^+^CD86^+^) cells in PANS flare compared to PANS recovery, and to healthy controls (Fig. 2A). In contrast, we found M2 (CD14^+^CD163^+^) monocytes were increased in patients in recovery (Fig. 2B). Thus, the proportion of each type of polarized monocyte subset al.igns with clinical status; this polarization may either reflect or contribute to PANS flare and recovery.

### Migratory monocytes and monocyte-derived dendritic cells are increased in PANS flare

**Fig. 3:**
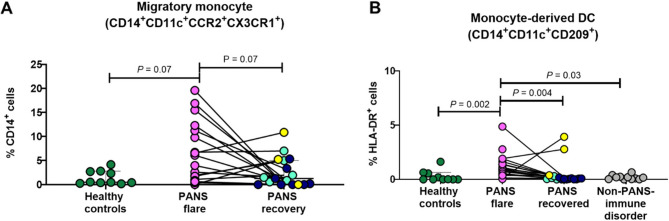
Migratory monocytes and monocyte-derived dendritic cells are increased in male patients with PANS in flare. Unstimulated PBMCs from male patients with PANS in flare (*n* = 17) and recovery (partial or full) (*n* = 17) (paired samples, *n* = 17) and age/sex(male)/race/ethnicity-matched healthy controls (*n* = 10) and “non-PANS immune disorder” controls (*n* = 11) were stained with marker panels defining “migratory monocytes” and “monocyte-derived dendritic cells”. **A** Proportion of “migratory monocytes” (CD14^+^CD11c^+^CCR2^+^CX3CR1^+^) are presented as a percent of CD14^+^ cells. The gates were set based on the fluorescence minus one (FMO) plot of CD11c, CCR2, and CX3CR1. **B** Monocyte-derived dendritic cells were gated and identified within HLA-DR^+^ cells as CD14^+^CD11b^+^CD11c^+^CD209^+^ population. CD209 gates were set based on the absence of CD209^+^ population within the CD16^+^ population; CD209 is not expressed by non-classical monocytes [72]. Paired samples are indicated by a straight line and were analyzed by Friedman Test (*P* value < 0.05 indicating significance). Unpaired samples were analyzed by non-parametric Mann-Whitney test (*P* value < 0.05 indicating significance). The different colors displayed in the recovery group represent three sub-groups of patients whose state and course (prior to their recovery) were characterized by: new-onset (light blue), flare-on-persistent baseline (dark blue) and static-persistent (yellow). “Non-PANS immune disorder” include non-PANS patients with other brain diseases or asthma

A novel circulating pro-inflammatory monocyte subset, defined by double positive, CCR2^+^CX3CR1^+^ cells, was recently described in patients with inflammatory bowel disease [66]. We looked for these pro-inflammatory migratory monocytes in PANS (Supplementary Fig. 1A) and found a population expressing the expected markers: CD14^+^CD11c^+^CCR2^+^ CX3CR1^+^ (Supplementary Table 11; Fig. 3A). These (likely) pro-inflammatory cells were elevated in male patients with PANS during flare and reduced during recovery (Fig. 3A). No changes were observed in female subjects (Supplementary Fig. 3A).

We next looked at the distribution of selected dendritic cell (DC) subsets. DCs, although typically derived from bone marrow precursors distinct from monocytes, can be generated from monocytes (mo-DC) in vitro by culture with GM-CSF and IL-4 [67]. Mo-DC generated in vivo in mice are marked by CD209 (DC-SIGN) [68]. The presence of circulating mo-DC in healthy individuals is debated [69], however, they have been identified in several diseases [70]. The proportion of circulating mo-DC (Supplementary Table 11, Supplementary Fig. 1B) was significantly increased in both male and female patients with PANS in flare compared to healthy controls and were typically decreased in recovery (Fig. 3B and Supplementary Fig. 3B). We also found that the proportion of the cDC1 subset of myeloid DC (CD14^-^CD16^-^CD11c^+^ HLA-DR^+^ CD141^+^; Supplementary Table 11), which functions in cross-presentation of exogenous antigens to CD8 T cells [71], was significantly increased in female, but not in male, patients with PANS in flare, compared to sex-matched healthy controls (Supplementary Fig. 4). CD141^+^ DC are required for the priming of protective CD8^+^ T cells and their presence may reflect prior infection or a role in PANS in post-infection. No differences in other DC subsets, including plasmacytoid DC and myeloid DCs, were observed between patients with PANS and healthy controls (not shown).

### “CNS-homing” CD14^+^ monocytes are reduced in the peripheral blood and present in the CSF in PANS flare

**Fig. 4:**
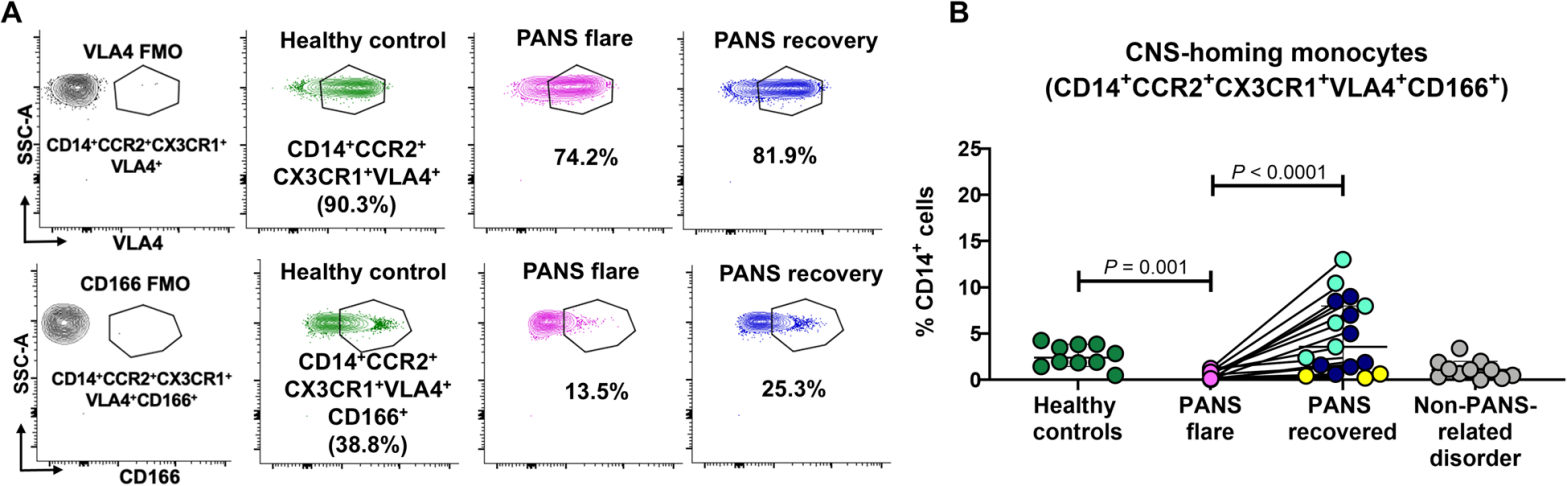
“CNS-homing” monocytes are reduced in circulation in PANS flare and increased in recovery (partial or full). Unstimulated PBMCs from male patients with PANS in flare (*n* = 17) and in recovery (partial or full) (*n* = 17) (paired samples = 17) and age/sex/race/ethnicity-matched healthy controls (*n* = 10) and “non-PANS immune disorder” controls (*n* = 11) were stained with a marker panel consistent with “CNS-homing.” **A** the gates were set based on the fluorescence minus one (FMO) plot of CCR2, CX3CR1, VLA4, and CD166. **B** Proportion of CD14^+^CCR2^+^CX3CR1^+^VLA4^+^CD166^+^ cells are presented as a percent of CD14^+^ cells. Paired samples are indicated by a straight line and were analyzed by Friedman Test (*P* value < 0.05 indicating significance). Unpaired samples were analyzed by non-parametric Mann-Whitney test (*P* value < 0.05 indicating significance). The different colors displayed in the recovery groups represent three sub-groups of patients whose state and course (prior to their recovery) were characterized by: new-onset (light blue), flare-on-persistent baseline (dark blue) and static-persistent (yellow). Non-PANS immune disorder includes non-PANS patients with brain diseases and asthma

**Fig. 5:**
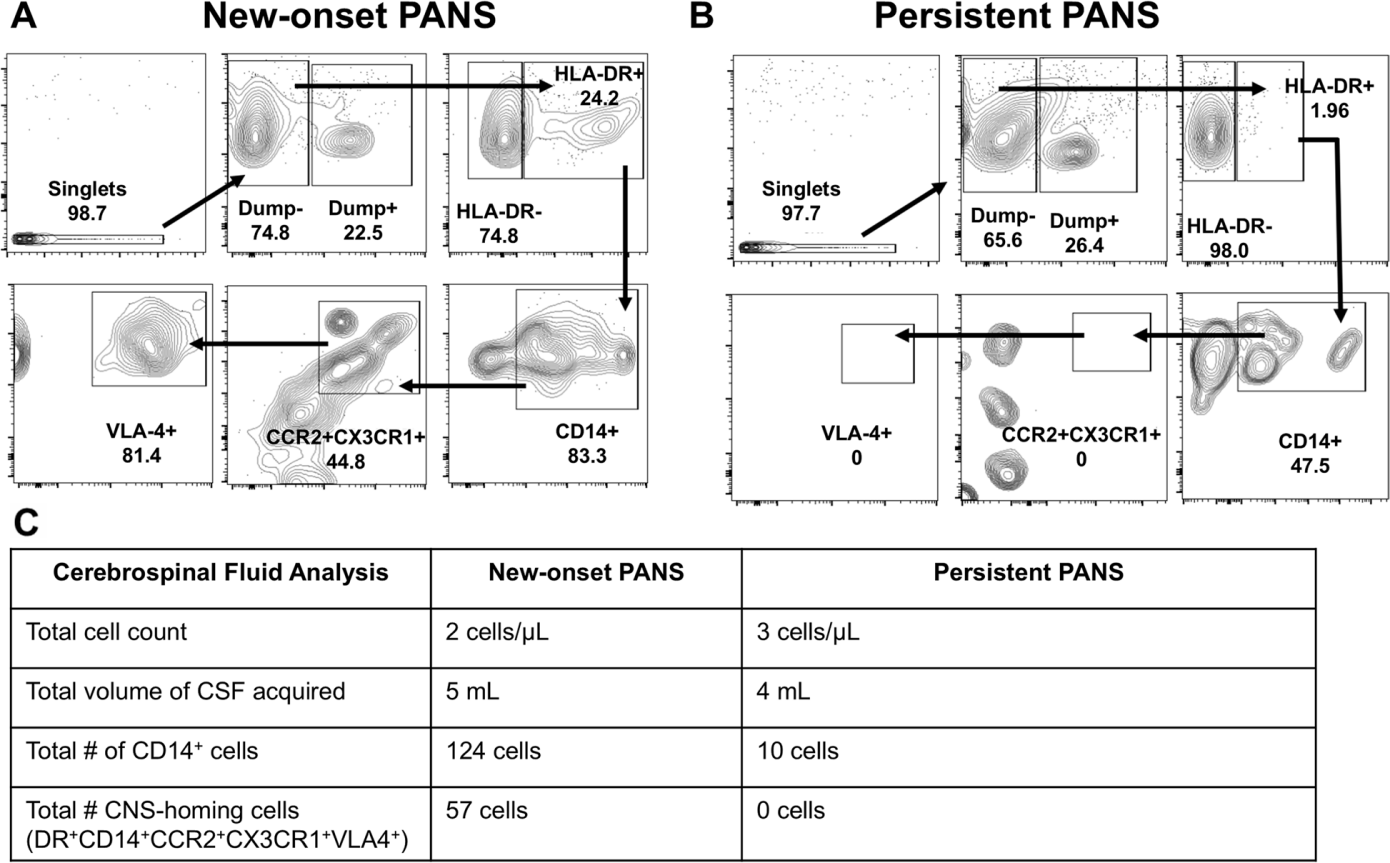
“CNS-homing” monocytes are present in the cerebrospinal fluid (CSF) of patients with new-onset PANS, but not patients with a persistent course of PANS. Cells were separated from CSF by centrifugation and stained to identify “CNS-homing” monocytes. Gating strategy to identify and count the number of “CNS-homing” monocytes in **A** a representative patient with new-onset PANS and **B** a representative patient on a persistent PANS course. **C** Quantification of CNS-homing monocytes per 5 mL CSF in patients with new-onset PANS (*n* = 7) and persistent PANS (*n* = 4). Data represent individual patients; horizontal bars denote mean ± SEM

The identification of both migratory monocyte and monocyte-derived dendritic cell subsets in the blood of patients with PANS suggests that these cells may be enrooted to areas of inflammation in the brain. To explore this possibility further, we aimed to detect specific monocytes that might be migrating towards the brain. These cells, which we refer to as “CNS-homing” monocytes, were characterized using a novel panel of markers (HLA-DR, CD14, CCR2, CX3CR1, VLA-4, CD166, Supplementary Fig. 1A and Fig. 4A) tailored to track their potential movement towards brain tissue. During PANS flare, the number of peripheral “CNS-homing” monocytes, identified by their expression of CD14, CCR2, CX3CR1, VLA4, and CD166, was significantly lower compared to healthy controls. As patients recovered, the proportion of these monocytes increased in the periphery (Fig. 4A, B).Notably, these “CNS-homing” monocytes were also detected in the cerebrospinal fluid (CSF) of patients with new-onset PANS (7 of 7 tested). Male gender, *n* = 7 (100%); age (4–15) years old; Fig. 5A-C) consistent with trafficking of these cells into the CNS during PANS flare. Interestingly, in patients with a persistent course of PANS (*n* = 4; male gender), these cells were at low frequency in the circulation (Fig. 4A, yellow group) and not detectable in the CSF (Fig. 5C).

### Intracellular cytokine profile of “CNS-homing” monocytes in PANS

Given their potential regulatory role in PANS, we evaluated the cytokine profile of “CNS-homing” monocytes. These were defined by the surface marker profile CD14⁺CCR2⁺CX3CR1⁺VLA-4⁺CD166⁺ and compared to “non-CNS-homing” monocytes (CD14⁺CCR2⁺CX3CR1⁻) across multiple clinical states. Using fixed whole blood samples, we measured the proportion of cytokine-positive cells and their median fluorescence intensities (MFI) (Supplementary Fig. 5, Table 8 and 9). Across all clinical states—flare, recovery, and healthy controls—these parameters remained stable within each monocyte subset (Supplementary Fig. 6). For example, both IL-10⁺ and TGF-β⁺ frequencies and MFI remained consistent in “CNS-homing” and “non-CNS-homing” monocytes across groups (Supplementary Fig. 6A–D), suggesting a stable cytokine signature.

**Fig. 6:**
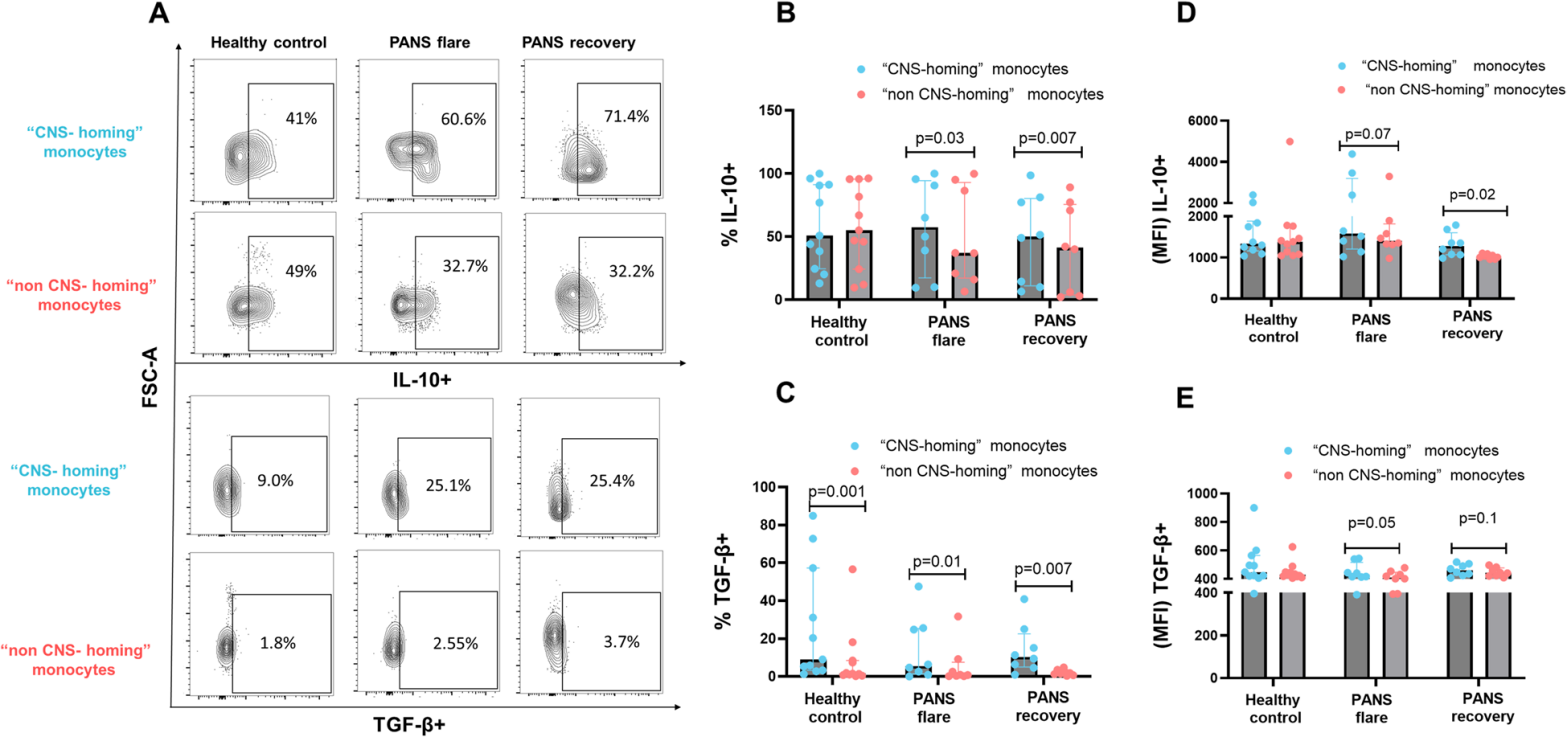
Intracellular production of IL-10, and TGF-β (Anti-inflammatory cytokines) in “CNS-homing” (blue) vs. “non-CNS-homing” (red) monocytes in PANS. Unstimulated smart tube whole fixed blood from patients with PANS in flare and in recovery (partial or full) (paired samples)(*n* = 8 each) and age/sex/race/ethnicity-matched healthy controls (*n* = 11) were stained with markers for intracellular cytokines measurements in “CNS-homing” vs. “non-CNS-homing” (CD14^+^CCR2^+^CX3CR1^-^) monocytes. **A** representative merged histograms for intracellular IL-10 and TGF-β measured by flow cytometry in “CNS-homing” vs “non-CNS-homing” monocytes. **B **and** C** Frequency IL-10^+^ and TGF-β^+^ (respectively) are presented as a percent of “CNS-homing” and “non- CNS-homing” monocytes. **D **and** E** MFI of IL-10^+^ and TGF-β^+^ (respectively). Data are represented as median with interquartile range.*p < 0.05, **p < 0.01, ***p < 0.001,****p < 0.0001; significant differences were determined using the nonparametric Wilcoxon’s test and the two sample Mann-Whitney test

When comparing the two subsets directly, “CNS-homing” monocytes exhibited significantly higher IL-10 expression, both in frequency and MFI (Fig. 6B, D), as well as an elevated frequency of TGF-β⁺ cells (Fig. 6C), though MFI was comparable (Fig. 6E). TGFB1, encoding TGF-β1, was also significantly enriched in “CNS-homing” monocytes by scRNA-seq analysis (adjusted *p* < 0.05, Wilcoxon test), ranking within the top 50 differentially expressed genes (Supplementary Fig. 7). IL10 RNA was not detected in the scRNA-seq dataset, likely due to technical limitations of droplet-based capture, rather than biological absence.

**Fig. 7:**
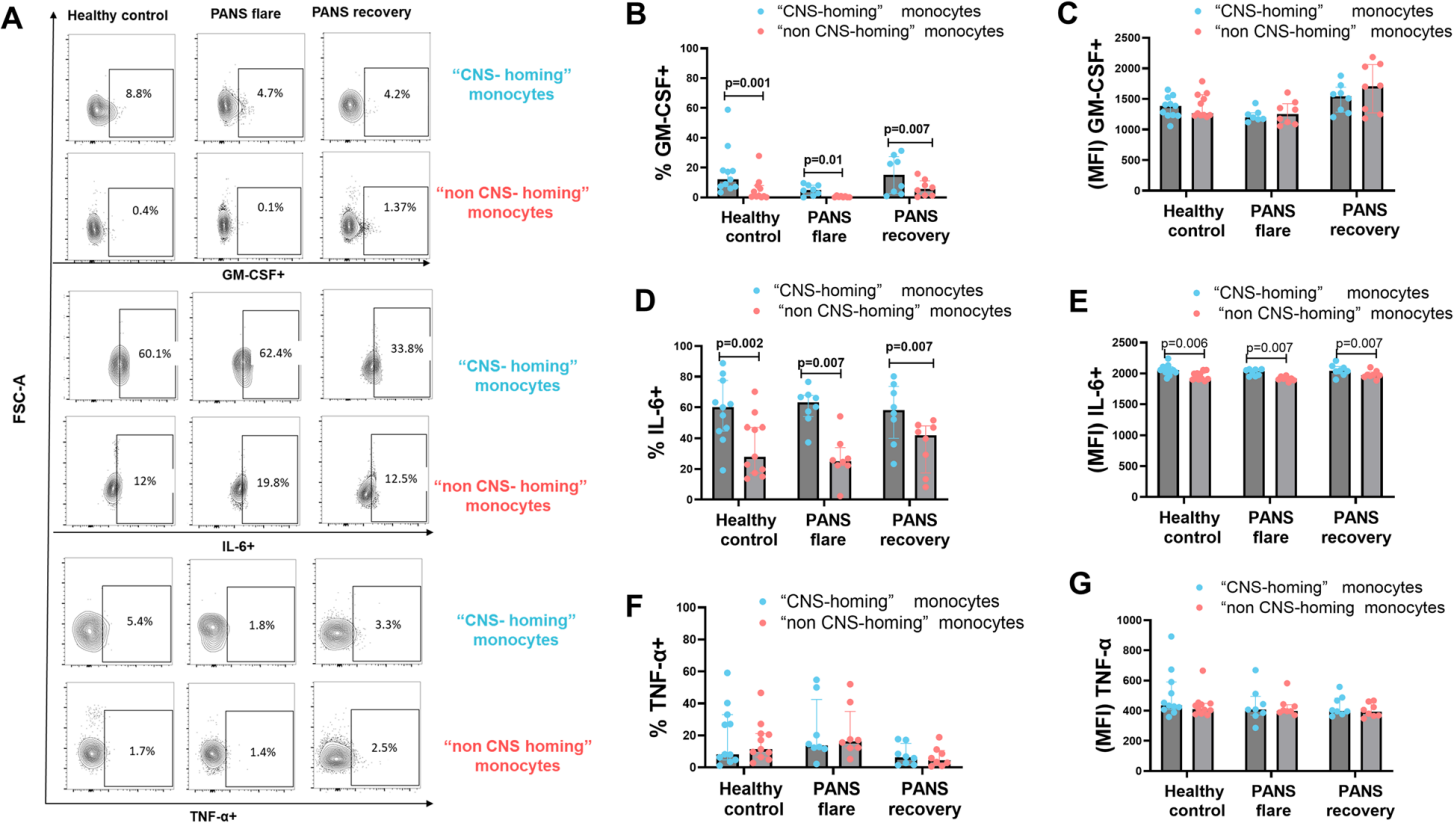
Intracellular production of GM-CSF, IL-6, and TNF-α in “CNS-homing” (blue) vs. “non-CNS-homing” (red) monocytes in PANS. Unstimulated smart tube whole fixed blood from patients with PANS in flare and in recovery (partial or full) (paired samples) (*n* = 8 for each) and age/sex/race/ethnicity-matched healthy controls (*n* = 11) were stained with markers for intracellular cytokines measurements in “CNS-homing” vs. “non-CNS-homing” monocytes. **A** representative merged histograms for intracellular GM-CSF, IL-6, and TNF-α measured by flow cytometry **B**,** D**,** F** Frequency of GM-CSF^+^, IL-6^+^, and TNF-α^+^ (respectively) are presented as a percent of “CNS-homing” vs “non- CNS-homing” monocytes. **C**,** E**,** G** MFI of GM-CSF^+^, IL-6^+^, and TNF-α^+^ (respectively) in the “CNS-homing” and “non-CNS-homing” monocytes. Data are represented as median with interquartile range. *p < 0.05, **p < 0.01, ***p < 0.001,****p < 0.0001; significant differences were determined using the nonparametric Wilcoxon’s test and the two sample Mann-Whitney test

We also assessed GM-CSF expression. While the frequency of GM-CSF⁺ cells was modestly higher in “CNS-homing” monocytes during recovery (Fig. 7B), MFI remained comparable between the two subsets (Fig. 7C). A modest drop in GM-CSF frequency was observed in both monocyte populations during flare (Supplementary Fig. 6), potentially reflecting disease-state influences.

For IL-6, “CNS-homing” monocytes showed a modest but statistically significant increase in frequency and MFI (Fig. 7D–E). Although only a small number of IL6-expressing cells were detected in the scRNA-seq data (*n* ≈ 10), the directionality of change aligned with the FACS trends. We acknowledge the limited power of these RNA-based results but present them as hypothesis-generating signals for future work. GM-CSF and IL10 transcripts were not detected in the scRNA-seq dataset, again likely due to technical constraints.

Lastly, TNF-α expression did not differ meaningfully between subsets (Fig. 7F–G), nor did TNF RNA levels in scRNA-seq (Supplementary Fig. 7). Altogether, these findings suggest that “CNS-homing” monocytes are enriched for anti-inflammatory and potentially neuro-reparative features. Their reduced frequency in patients with a persistent disease course raises the possibility that these cells may play a protective role during recovery, warranting further investigation.

### Plasma from relapsing-remitting PANS induces “CNS-homing” markers in healthy monocytes

**Fig. 8:**
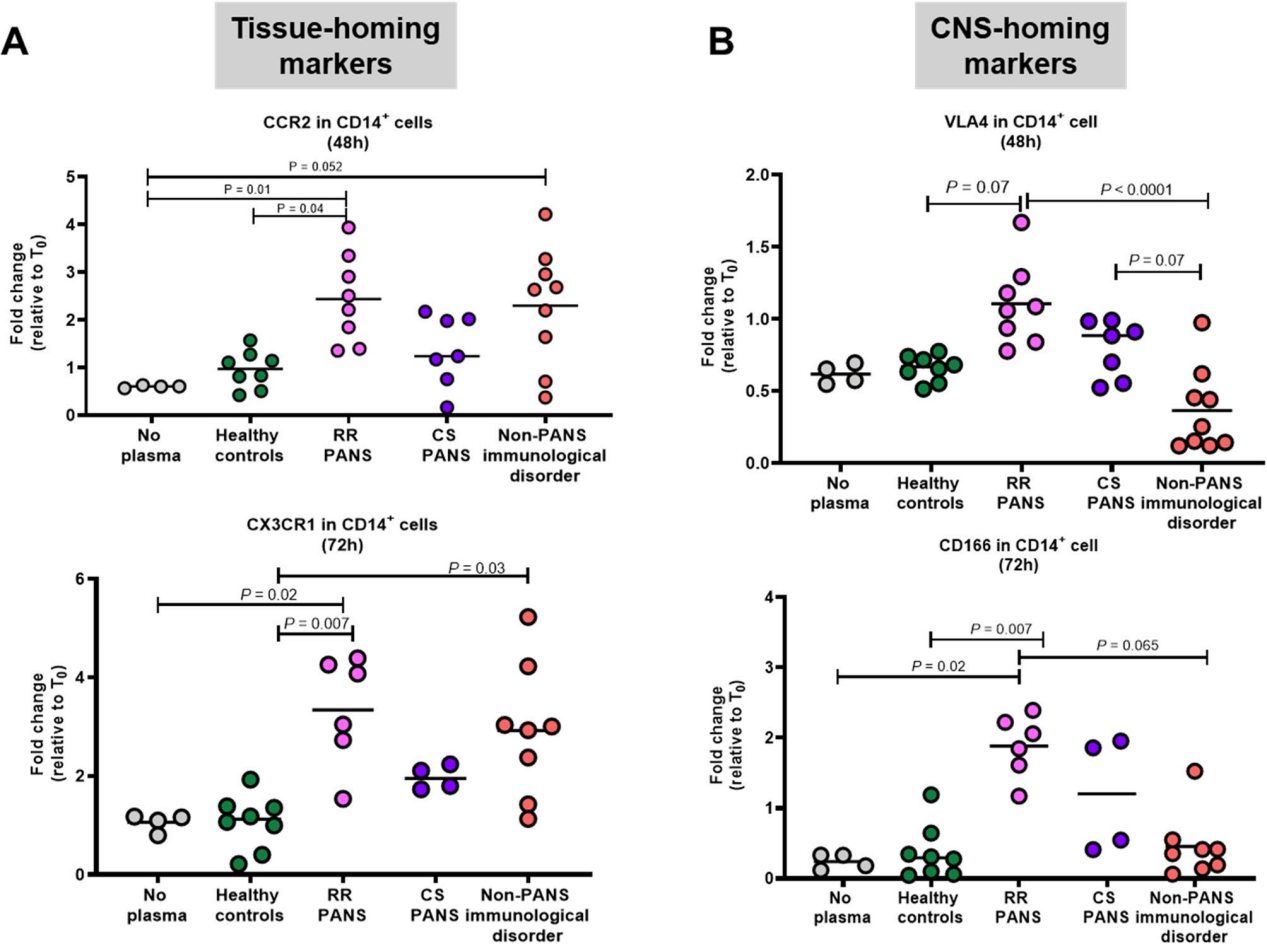
Plasma from relapsing-remitting PANS induces “CNS-homing” markers in healthy monocytes. Peripheral blood mononuclear cells were isolated from a healthy donor and exposed to 5% plasma from patients with a relapsing-remitting course of PANS (in flare), patients with a persistent course of PANS (CS), age/sex/race/ethnicity matched healthy donors, and non-PANS immune disorder controls (juvenile arthritis, asthma) disease controls for 0–72 h. Fold changes in the median fluorescent intensity of **A** of CCR2 and CX3CR1 (tissue-homing markers in monocytes) and **B** VLA4 and CD166 (“CNS-homing” markers in monocytes) at time 48-h and 72-h are plotted relative to time 0 (T0)

The regulation of monocyte trafficking and function is often driven by circulating factors [42]. To begin to explore this possibility for the ‘’CNS-homing’’ monocytes, we exposed healthy donors’ monocytes to PANS plasma and to plasma from inflammatory non-PANS disorders. We found that both types of plasma induce the tissue-homing chemokine receptors, CCR2 and CX3CR1 that mediate trafficking of monocytes to sites of inflammation (Fig. 8A). However, the “CNS-homing” markers (VLA-4, CD166) expression were uniquely induced by the PANS plasma (Fig. 8B), suggesting the possibility that circulating factors may influence monocyte localization signatures, although this remains to be validated. These experiments were exploratory in nature and intended to generate preliminary insights into how circulating factors in PANS plasma may modulate monocyte surface marker expression. While these findings suggest the presence of plasma-derived signals influencing CNS-homing phenotypes, further mechanistic work is needed to identify and validate these signals.

## Discussion

Pediatric Acute-onset Neuropsychiatric Syndrome is a complex disorder, causing a range of neuropsychiatric symptoms in children. The etiology of PANS is not well understood, although evidence is accumulating for a key role of immune dysregulation [6]. This includes the finding of antibodies to cholinergic interneurons in both PANS and a subset of PANS, termed PANDAS (pediatric autoimmune neuropsychiatric disorders associated with streptococcus), arguing for a contribution of adaptive immunity to pathogenesis [27]. Previous PANDAS studies suggested the role of anti-neuronal antibodies to basal ganglia, a CNS region implicated in motor and other behavioral abnormalities exhibited by patients with PANDAS [29, 33, 73–75]. Other studies showed that PANS is associated with neuroinflammation of the basal ganglia [18] and infiltration of autoantibodies induced by infection to the CNS from the circulation to target dopamine receptor [34, 76]. Here, we report changes in frequencies of monocyte and dendritic cell subsets associated with changes in clinical phenotype in PANS. Our findings offer strong evidence for a role of innate immune responses in this disease.

A key finding of this study is the identification of a subset of CD14 + monocytes with the potential to home to the brain, based on their expression of selected markers, a subset we named “CNS-homing” monocytes. During flare, “CNS-homing” monocytes are reduced in frequency in blood and detected in the CSF, arguing for their potential migration to the brain. However, we acknowledge that the detection of monocytes in the CSF does not directly imply disruption of the BBB or parenchymal invasion. Rather, these cells may access the CSF via the fenestrated choroid plexus, a component of the blood-CSF barrier. Additional anatomical compartments, such as the perivascular space, may also serve as reservoirs for CD14^+^CD163^+^ monocytes in neuropsychiatric conditions [77]. Future studies will be necessary to determine the precise anatomical destinations and functional roles of these cells in the CNS.

It is interesting to point out that ‘’CNS-homing’’ cells were at low frequency in the circulation of patients with persistent PANS and not detectable in the CSF of 4 patients with persistent PANS. It might be possible that in the ‘persistent’ course of the disease, circulating factors play a minor role. Although we do not currently have any supportive evidence, it is possible that “trained innate immunity” and the epigenetic changes of the innate cells may contribute to the pathogenesis of persistent PANS [78].

Our marker expression analysis showed a reduction of the chemokine receptor CCR2, and elevation of CX3CR1 during PANS flare. These results suggest possible CX3CR1-dependent trafficking of the “CNS-homing” monocytes. Of note, recent work by Cugurra et al. has shown that bone marrow niches in the murine skull can also give rise to immune cells in the CSF, with distinctive transcriptional profiles [79]. The possibility of a similar finding in human neuroinflammatory disorders remains to be investigated.

The presence of the ‘’CNS-homing” subset in the cerebrospinal fluid of patients with PANS signifies its potential trafficking to the brain. It would be important to know if these cells reside in the meninges or actually traffic to specific brain regions. However, obtaining human brain tissue is difficult, making this one of the limitations of the study.

Future studies, including potential animal models, are warranted to study the infiltration of CNS with monocytes in PANS. Exactly how myeloid cells like monocytes infiltrate the CNS parenchyma remains to be established, but it is plausible that the BBB, consisting of tight junctions, may be disrupted under neuroinflammatory conditions [80], such as during PANS flare. Indeed, our lab data show leakiness in a BBB in vitro model using human primary brain endothelial cells exposed to PANS plasma during flare (Mondal et al., manuscript in preparation).

Our analysis of the intracellular cytokine profiles suggests that the “CNS-homing” monocytes, distinguished by their expression of VLA-4 and CD166, have an anti-inflammatory profile compared to the “non-CNS-homing” monocytes in patients with PANS. In particular, the “CNS-homing” monocytes produced significantly higher amounts of IL-10. A previous study showed that IL-10 producing CD14^+^ monocytes in the periphery have a regulatory role in neuroinflammatory diseases [81]. It’s interesting to point out a bimodal distribution in the proportion of IL-10 + in “non-CNS-homing” monocytes. While the percent is useful for determining the proportion of cells expressing the IL-10, it can be misleading if the intensity of expression within the positive population varies significantly. However, we observed a normal distribution in the mean fluorescence intensity (MFI) of IL-10^+^ in the “non-CNS-homing” monocytes despite the observed bimodality in proportion of IL-10, suggesting that it is the percentage of cells producing IL-10, not the expression, that is the key factor.

Furthermore, we show that “CNS-homing” monocytes produced more transforming growth factor-β (TGF-β) compared to the “non-CNS-homing” monocytes. Notably, TGF-β has established neuroprotective function and powerful anti-inflammatory properties [82]. TGF- β RNA expression confirms the trend observed in Fig. 6D with an increase of expression in both PANS flare and PANS recovery “CNS-homing” populations and a less significant and opposite change in the control samples.

Evidence from studies of experimental autoimmune encephalomyelitis (EAE) and multiple sclerosis patients showed that GM-CSF promotes the migration of monocytes to the CNS [83]. In agreement with this finding, our data showed that the “CNS-homing” monocytes subset produced more GM-CSF compared to the “non-CNS-homing” monocytes. The increase in GM-CSF in the “CNS-homing” and “non-CNS-homing” monocytes during PANS recovery appears to support the notion that higher GM-CSF levels during recovery might facilitate the movement of monocytes into the brain, which could play a role in the recovery process. This is consistent with prior research, highlighting GM-CSF as a key mediator in immune cell trafficking and CNS involvement during inflammatory or recovery phases [83]. These findings suggest the promotion of anti-inflammatory response. It is interesting to point out that our lab data showed the promotion of regulatory T (t_reg_) cells during PANS flare with a trend of decrease during recovery (Hussein et al., manuscript in preparation). It is plausible to think that the observed changes in the cytokines profile and dysregulations of t_reg_ cells could signify that the body is trying to combat the ongoing neuroinflammation in PANS.

Although IL-6 is traditionally classified as a pro-inflammatory cytokine, it can induce an anti-inflammatory cytokine profile, enhancing IL-4/IL-10 production, and polarize human monocytes into an anti-inflammatory M2 phenotype under certain co-stimulatory conditions [84, 85]. A number of findings suggest that IL-6 can counteract inflammatory responses and has protective functions during severe states of disease [86]. A recent mouse study showed that brain infiltrating CCR2^+^ monocytes (producing IL-6) drove repair of brain vasculature after cerebrovascular injuries [87]. Our data showed that the “CNS-homing” monocytes produced more IL-6 compared to the “non-CNS-homing” monocytes, suggesting that in PANS, IL-6 might serve a protective or reparative function. These findings suggest that “CNS-homing” monocytes have an immunosuppressive role confirmed by the scRNA-seq data, participating in a peripheral immune response to neuroinflammation. Specifically, these monocytes, which exhibit immunosuppressive properties, are responsive to brain-produced chemokines like fractalkine, known to be secreted by neurons [88].

Our current hypothesis that some monocytes adopt an anti-inflammatory state contributing to PANS recovery is further supported by the shift towards an M2-polarized phenotype as symptoms abate. M2-type cells have been shown to be superior in mobility compared to M1-type cells [89]. M2-type CD14^+^ cells may be trafficking to the brain to assist in the resolution of inflammation. In our previous study [39], we found that IVIG reduced the frequency of pro-inflammatory monocytes in PANS. Although not all patients in this cohort were treatment-naïve, efforts were Made to limit treatment-related confounding. None of the patient in this study had received IVIG within 3 months, and corticosteroid exposure was limited to 2 individuals. Moreover, by prioritizing paired flare-recovery samples, we aimed to reduce intra-individual variability and improve interpretability of immune shifts across disease states. Nonetheless, we acknowledge that NSAIDs and disease-modifying agents such as hydroxychloroquine or mycophenolate could influence monocyte polarization or cytokine profiles. These limitations are inherent to human observational studies but underscore the need for future work in treatment-stratified or treatment-naïve cohorts to dissect endogenous immune dynamics from pharmacologic effects.

Monocytosis, an increase in the total count of circulating monocytes, is a feature of many inflammatory disorders [90], including PANS [2]. In our study, male patients exhibited clear monocytosis during PANS flare relative to healthy male controls, which generally resolved during recovery. In contrast, patients on a persistent disease course displayed sustained monocytosis even in the presence of partial symptom improvement. In males, this monocytosis was primarily driven by an increase in classical (CD14⁺) monocytes, suggestive of an amplified innate immune response. Although monocytosis was less pronounced in female patients, most showed a reduction in total monocyte count during recovery. Notably, compared to their sex-matched healthy controls, female patients demonstrated an increase in intermediate monocytes during flare, whereas male patients exhibited a decrease in this subset.

These sex-associated patterns in monocyte subset dynamics may reflect fundamental biological differences in immune regulation. In males, the dominance of classical monocytes and persistent monocytosis could indicate a sustained pro-inflammatory state or impaired immune resolution. In contrast, the transient rise and normalization of intermediate monocytes in females may suggest a more controlled or regulatory immune trajectory. These observations are consistent with broader immunological trends in which females tend to mount more robust and coordinated immune responses [91], while males are often predisposed to exaggerated innate inflammatory reactivity [92]. This sexual dimorphism is influenced by hormonal factors, including the immunostimulatory effects of estrogen and the immunosuppressive role of testosterone, as well as by gene regulation linked to the X and Y chromosomes [93]. Sex-based immune dimorphism is increasingly recognized in neurodevelopmental disorders, including ASD [94] and autoimmune encephalitis [95]. Our data extend this concept to PANS, revealing both overlapping and divergent monocyte responses between male and female patients.

Sex differences in monocyte subset frequency have been observed in other studies [96]. Interestingly, analysis of human immune cell proportions of almost 800 healthy individuals (Milieu Interieur Consortium) showed that, among young subjects, males had significantly higher monocyte percentages than females and that the proportion of monocytes increases with age in females [64]. Male mice have a larger splenic reservoir of classical monocytes than females, resulting in more pronounced monocytosis [97]. These findings raise the possibility that, compared to females, male patients with PANS release more monocytes into the circulation during flare. Another explanation may be that males have more externalizing behaviors (aggression and other challenging behaviors) compared to females and thus are likely to be referred earlier to our clinic and have their first blood draw at an earlier stage than females [14]. Overall, our data suggest both distinct and shared immune pathways may contribute to PANS pathogenesis in male and female patients.

Our data on dendritic cell populations in PANS revealed that flares are associated with increased monocyte-derived DC (Mo-DC) frequency. Mo-DC are a distinct DC subset, originating upon stimulation of circulating monocytes; they are involved in inflammation and infection in autoimmune diseases [98]. To our knowledge, the detection of Mo-DC has not been reported previously in neuroinflammatory disease. Their presence in the circulation of patients with PANS in flare argue that they contribute to disease, perhaps by producing reactive oxygen species (ROS) via the JAK/STAT axis, as observed in inflammatory arthritis [98]. It will be of interest to identify factors in plasma that induce the development of Mo-DC in PANS. Most tissue-resident macrophages are derived from erythro-myeloid progenitors, seeded from the yolk sac [99]. However, circulating monocytes also can differentiate into tissue macrophages [89].

We observed a striking difference in the proportion of CD141^+^ DCs in our female patient cohort. CD141^+^ DCs are potent antigen presenting myeloid cells that are known to induce CD4^+^ T cells and stimulate the production of type 2 cytokines. Although there is limited evidence of the role of CD141^+^ DCs in neuroinflammation in the literature, our data indicates a potential role of these cells in the pathogenesis of PANS, particularly in female patients. Given that CD141⁺ DCs are key producers of IL-12 and cross-present antigens, their increase in recovery may reflect a role in immune recalibration or neurorepair in female, though this remains speculative in the context of PANS. While this study focused on monocytic subsets, it will be interesting to evaluate the function and behavior of DC subsets in future studies.

### Limitations of our study

While our study provides new insights into innate immune dynamics in PANS, several considerations warrant attention. First, as a pilot investigation, the sample size is modest and likely reflects clinical heterogeneity inherent to the PANS population. While this limits statistical power for certain subgroup analyses, it also highlights the need for multi-site efforts and larger cohorts in future work.

Although we identified monocyte populations with a surface phenotype consistent with CNS-homing and detected these cells in CSF, our conclusions about migration remain indirect. We did not perform in vivo imaging or trafficking assays, and thus refer to these cells as having a “CNS-homing phenotype” rather than definitively classifying them as brain-infiltrating. Future animal models and in vivo tracking studies will be essential to validate this migratory behavior.

In our single-cell RNA-seq analysis, the small number of PANS samples precluded the use of compositional modeling tools such as MASC, scCODA, or Milo. We instead reported observed trends in subset distribution as exploratory and emphasize that these findings are hypothesis-generating pending validation in larger scRNA-seq cohorts.

Finally, the plasma exposure experiments were intentionally designed as a preliminary step to assess whether circulating factors during PANS flare could modulate monocyte surface phenotype. While the results support this possibility, they do not permit mechanistic conclusions. Follow-up studies isolating specific plasma components or mediators will be needed to elucidate causal pathways and their relevance to in vivo immune programming.

PANS is a pediatric disease of unknown etiology with substantial morbidity for affected children and high caregiver burden for their families [100]. Current treatment strategies alleviate symptoms in many patients, but relapses are common. Our identification of distinct monocyte subsets associated with flare and recovery highlights a potentially targetable arm of the innate immune system in PANS. Although monocytes comprise a relatively small fraction of circulating leukocytes, their contributions to neuroimmune pathology are increasingly recognized, both through direct effector functions and their differentiation into tissue macrophages [101]. In neuroinflammation, monocytes have been shown to infiltrate the brain and contribute to disease pathology [102]. These subsets may serve as biomarkers of disease trajectory or therapeutic response. Ultimately, elucidating how immunosuppressive or reparative monocyte states are induced—endogenously or by treatment—may guide precision therapies to reduce relapse burden and improve long-term outcomes for affected children.

## Supplementary Information

Supplementary Material 1.

Supplementary Material 2.

## Data Availability

No datasets were generated or analysed during the current study.
