# Supplementary material for: Sex-associated and disease state-dependent monocyte polarization and CNS-trafficking phenotypes in pediatric acute-onset neuropsychiatric syndrome (PANS)
Source: J Neuroinflammation. 2025 Nov 18;22:273. doi: 10.1186/s12974-025-03549-6 (PMC12625707; doi:10.1186/s12974-025-03549-6)
Supplement: Supplementary file 1 — Supplementary Material 1. [file 12974_2025_3549_MOESM1_ESM.docx]

**Supplementary data**

**Disease state-dependent myeloid cell phenotypes in pediatric acute-onset neuropsychiatric syndrome including a novel “CNS-homing” monocyte subset**

**Authors:** Shamma S. Rahman^1^*^, Noor Hussein^1^*^, Silvia Giulia Galfrè^2^ Fabian Gaertner^1^, Claudia Macaubas^1^, Avis Chan^3,4^, Laurie Columbo^3,4^, Jaynelle Gao^3,4^, Samira Galehdari^1^, Batuhan Bayram^1^, Mei Ma^3,4^, Cindy Manko^3,4^, Kate Miles^3,4^, Bahare Farhadian^3,4^, Melissa Silverman^4,5^, Margo Thienemann^4,5^, Noga Or-Geva^6^, Keith Van Haren^6^, Kari C. Nadeau^3^, Lu Tian^7^, Jennifer Frankovich^3,4^*^, Elizabeth D. Mellins^1^#^

**Supplementary Table 1**. **Definitions of PANS disease state and disease course**(adapted from Masterson et al. 2025[4, 22], Table 2 and Figure 3)

| **Term** | **Definition** |
| --- | --- |
| **Baseline impairment level** | a patient’s level of functioning prior to their initial neuropsychiatric flare, which, for some patients, may include symptoms related to other conditions such as ADHD, ASD, etc. |
| **Patient Status (disease state)** |  |
| **In flare** | in a state of escalated symptoms and heightened functional impairment relative to a patient’s baseline impairment level    *Note 1: this does* not *include minor symptom escalations (see below)*  *Note 2: this includes flare(s) on good baseline and flare(s) on persistent disease course. See definition of persistent course below.* |
| Isolated flare | the occurrent of one flare start, which ends with an unequivocal recovery |
| Flare series | the consecutive occurrence of at least two flare starts without an unequivocal recovery between; a notable exacerbation of symptoms and/or an escalation in new symptoms (i.e., a new flare) that occurs during an ongoing flare |
| **In recovery** | in a state of improvement regarding symptoms and function relative to the peak symptoms of the most recent flare    *Note: in our database, an unequivocal recovery from escalated symptoms and heightened functional impairment indicates the end of a flare* |
| Partial recovery | unequivocally recovered from a flare, but with residual functional deficits from the prior flare(s) that are above their baseline impairment level    *Note: a partially recovered patient may eventually reach a “full” recovery* |
| Full recovery | unequivocally recovered from a flare; symptoms and functional impairment are equivalent or near equivalent to patient’s baseline impairment level |
|  |  |
| **Minor symptom escalation** | a slight increase in neuropsychiatric symptoms that does not drastically increase patient functional impairment    *Note: these samples were not used for this myeloid cell project* |
| **Clinical Course**  **(disease course)** |  |
| **Single Flare** | Characterized by one recorded flare to date |
| **Relapsing-Remitting** | Characterized by intermittent flare and recovery intervals; has had at least one unequivocal recovery and subsequent flare start |
| **Primary Persistent** | Characterized by an initial flare (isolated or flare series) that lasts >12 months without an unequivocal recovery |
| **Secondary Persistent** | Characterized by a subsequent flare (isolated or flare series) that lasts >12 months without an unequivocal recovery |

**Supplementary Table 2:** Clinical data on patients with PANS* who were in the monocyte experiments (Tables 1 and 2 and Figures 1, 3 and 4).

|  | **Patients**  (n=36**) | **Controls**  (n=20) |
| --- | --- | --- |
| Age at the first clinic visit, mean (SD), y | 11.2 (3.9) | - |
| Age at first neuropsychiatric decline, mean (SD), y | 8.6 (2.9) | - |
| Male gender, n (%) | 18 (50) | 10 (50) |
| Non-Hispanic, n (%) | 32 (89) | 18 (90) |
| Age at blood draw, mean (SD), y | 12.3 (4.1) | 13.0 (5.8) |
| Treatments at time of blood draw, n (%)*** |  |  |
| NSAIDs | 18 (49) | - |
| Hydroxychloroquine | 5 (14) |  |
| Mycophenolate mofetil | 2 (5) | - |
| Treatments leading up to blood draw, n (%)*** |  |  |
| Corticosteroids (oral) in prior 4 weeks | 2 (5) | - |
| Corticosteroids (IV) in prior 4 weeks | 0 (0) | - |
| IVIG in prior 3 months | 0 (0) | - |
| Non-medicated, n (%) | 6 (16) | - |

* At the time of the selection of samples, all patients were classified as PANS. As part of an audit, and more rigorous classification of patients, one patient was not a confirmed PANS case (due to lack of outside records confirming acute-onset).

**One patient contributed one sample to the new-onset subset experiment and another sample to a separate experiment while on a persistent course.

***Flare samples were from patients not on any immunomodulation except for NSAIDs in some. Patients in the recovery state were often on immunomodulators (corticosteroids, IVIG, hydroxychloroquine, and mycophenylate mofetil).

Abbreviations: IV= Intravenous, IVIG= Intravenous immunoglobulin

**Supplementary Table 3**: Clinical data on patients with PANS who were in the Polarized Monocyte (Macrophage-like cells) experiments (Figure 2).

|  | **Patients**  (n=23) | **Controls**  (n=26) |
| --- | --- | --- |
| Age at the first clinic visit, mean (SD), y | 11.7 (3.7) | - |
| Age at first neuropsychiatric decline, mean (SD), y | 9.6 (3.4) | - |
| Male gender, n (%) | 16 (70) | 15 (58) |
| Non-Hispanic, n (%) | 23 (100) | 20 (77) |
| Age at blood draw, mean (SD), y | 14.3 (4.3) | 14.1 (3.7) |
| Treatments at time of blood draw, n (%)*** |  |  |
| NSAIDs | 12 (52) | - |
| Hydroxychloroquine | 2 (9) | - |
| Methotrexate | 1 (4) | - |
| Treatments leading up to blood draw, n (%)*** |  |  |
| Corticosteroids (oral) in prior 4 weeks | 1 (4) | - |
| Corticosteroids (IV) in prior 4 weeks | 1 (4) | - |
| IVIG in prior 3 months | 2 (9) | - |
|  |  |  |
|  |  |  |
| Non-medicated, n (%) | 5 (22) | - |

***Flare samples were from patients not on any immunomodulation except for NSAIDs in some. Patients in recovery were often on immunomodulators (steroids, IVIG, hydroxychloroquine, and methotrexate).

Abbreviations: IV= Intravenous, IVIG= Intravenous immunoglobulin

**Supplementary Table 4:** Clinical data on patients with PANS (cytokines study in Figures 6 and 7).

|  | **Patients**  (n=8) | **Controls**  (n=11) |
| --- | --- | --- |
| Female gender, n(%) | 3(37.5) | 7(63.6) |
| Male gender, n (%) | 5(62.5) | 6 (54.5) |
| Age at the time of blood draw, mean (SD), y | 12.58 (3.7) | 13.1 (3.7) |
| Treatments at time of blood draw, n (%)*** |  |  |
| NSAIDs | 2(25) | - |
| Maintenance immunomodulators | 3(37.5) | - |
| Hydroxychloroquine | 0 (0) | - |
| Methotrexate | 0 (0) | - |
| Treatments leading up to blood draw, n (%)*** |  |  |
| Corticosteroids (oral) in prior 4 weeks | 4 (50) | - |
| Corticosteroids (IV) in prior 4 weeks | 0 (0) | - |
| IVIG in prior 3 months | 1 (12.5) | - |
| Non-medicated, n (%) | 1(12.5) | - |

***Flare samples were from patients not on any immunomodulation except for NSAIDs in some. Patients in recovery were often on immunomodulators (steroids, IVIG, hydroxychloroquine, and methotrexate).

Abbreviations: IV= Intravenous, IVIG= Intravenous immunoglobulin

| **Supplementary Table 5:** **Marker panel for identifying “CNS-homing” monocytes** | | | | |
| --- | --- | --- | --- | --- |
| **Marker** | **Fluorophore** | **Clone** | **Vendor** | **Cat.No** |
| AQUA (Live/dead) | Aqua |  | Thermo Fisher Scientific Inc. | L34957 |
| CD3, CD19, CD56, CD66b | PerCPCy5.5 | HIT3a, HIB19, [MEM-188](https://www.biolegend.com/fr-ch/search-results?Clone=MEM-188), [G10F5](https://www.biolegend.com/fr-ch/search-results?Clone=G10F5) | BioLegend® | 300328, 302230, 304626, 305108 |
| HLA-DR | APC Cy7 | [L243](https://www.biolegend.com/en-us/search-results?Clone=L243) | BioLegend® | 307618 |
| CD14 | Pacific Blue | [HCD14](https://www.biolegend.com/en-us/search-results?Clone=HCD14) | BioLegend® | 325616 |
| CD16 | BV650 | 3G8 | BioLegend® | 302042 |
| CCR2 | BV605 | K036C2 | BioLegend® | 357214 |
| CX3CR1 | APC | 2A9-1 | BioLegend® | 341610 |
| CD68 | PE Cy7 | Y1/82A | BioLegend® | 333816 |
| Iba1 | FITC | Ab15691 | ABCAM | 1022-5 |
| Vla4 | PEDazzle | 9F10 | BioLegend® | 304326 |
| CD11c | BV711 | 3.9 | BioLegend® | 301630 |
| CD166 (ALCAM) | PE | 3A6 | BioLegend® | 343904 |

| **Supplementary Table 6: Marker panel for identifying dendritic cells** | | | | |
| --- | --- | --- | --- | --- |
| **Marker** | **Fluorophore** | **Clone** | **Vendor** | **Cat.No** |
| AQUA (Live/dead) | Aqua |  | Thermo Fisher Scientific Inc. | L34957 |
| CD3, CD19, CD56, CD66b | PerCPCy5.5 | HIT3a, HIB19, [MEM-188](https://www.biolegend.com/fr-ch/search-results?Clone=MEM-188), [G10F5](https://www.biolegend.com/fr-ch/search-results?Clone=G10F5) | BioLegend® | 300328, 302230, 304626, 305108 |
| HLA-DR | APC Cy7 | [L243](https://www.biolegend.com/en-us/search-results?Clone=L243) | BioLegend® | 307618 |
| CD14 | Pacific Blue | [HCD14](https://www.biolegend.com/en-us/search-results?Clone=HCD14) | BioLegend® | 325616 |
| CD16 | BV650 | 3G8 | BioLegend® | 302042 |
| CD11b | BV605 | ICRF44 | BioLegend® | 301332 |
| CD11c | BV711 | 3.9 | BioLegend® | 301630 |
| CD1c | FITC | L161 | BioLegend® | 331518 |
| CD141 | APC | M80 | BioLegend® | 344106 |
| CD123 | PE CY5 | 6H6 | BioLegend® | 306008 |
| CD83 | BV785 | HB15E | BioLegend® | 305338 |
| CD209 (DC-SIGN) | PE | 9E9A8 | BioLegend® | 330106 |

| **Supplementary Table 7:** **Marker panel for identifying circulating macrophage-like cells** | | | | |
| --- | --- | --- | --- | --- |
| **Marker** | **Fluorophore** | **Clone** | **Vendor** | **Cat.No** |
| AQUA (Live/dead) | Aqua |  | Thermo Fisher Scientific Inc | L34957 |
| CD3, CD19, CD56, CD66b | PerCPCy5.5 | HIT3a, HIB19, [MEM-188](https://www.biolegend.com/fr-ch/search-results?Clone=MEM-188), [G10F5](https://www.biolegend.com/fr-ch/search-results?Clone=G10F5) | BioLegend® | 300328, 302230, 304626, 305108 |
| HLA-DR | APC Cy7 | [L243](https://www.biolegend.com/en-us/search-results?Clone=L243) | BioLegend® | 307618 |
| CD14 | Pacific Blue | [HCD14](https://www.biolegend.com/en-us/search-results?Clone=HCD14) | BioLegend® | 325616 |
| CD16 | PE Cy7 | 3G8 | BioLegend® | 302016 |
| CD64 | PEDazzle | 10.1 | BioLegend® | 305032 |
| CD86 | BV605 | BU63 | BioLegend® | 374214 |
| IL-1b | FITC | JK1B-1 | BioLegend® | 508206 |
| CD163 | APC | GH1/61 | BioLegend® | 333610 |
| CD206 | PE | 15-2 | BioLegend® | 321106 |

| **Supplementary Table 8: Marker panel for evaluating intracellular cytokines** | | | |
| --- | --- | --- | --- |
| **Marker** | **Fluorophore** | **Clones** | **Vendor** |
| CD235a,CD15, CD3, CD19, CD56, CD66b | PerCPCy5.5 | [HI264](https://www.biolegend.com/fr-ch/search-results?Clone=HI264), [HI98](https://www.biolegend.com/fr-ch/search-results?Clone=HI98),HIT3a,  HIB19, [MEM-188](https://www.biolegend.com/fr-ch/search-results?Clone=MEM-188), [G10F5](https://www.biolegend.com/fr-ch/search-results?Clone=G10F5) | BioLegend®  BioLegend® |
| HLA-DR | APC Cy7 | [L243](https://www.biolegend.com/en-us/search-results?Clone=L243) | BioLegend® |
| CD14 | Pacific Blue | [HCD14](https://www.biolegend.com/en-us/search-results?Clone=HCD14) | BioLegend® |
| CD16 | BV650 | 3G8 | BioLegend |
| CCR2 | BV605 | K036C2 | BioLegend® |
| CX3CR1 | BV785 | 2A9-1 | BioLegend® |
| VLA-4 | BV510 | 9F10 | Biolegend® |
| CD166 | PE | 3A6 | Biolegend® |
| TNF-a | BV711 | MAb11 | Biolegend® |
| IL-6 | FITC | MQ2-13A5 | Biolegend® |
| TGF-β | PE-CF594 | TW4-2F8 | Biolegend® |
| IL-10 | PE cy7 | JES3-9D7 | Biolegend® |
| GM-CSF | APC | BVD2-21C11 | Biolegend® |
|  |  |  |  |

| **Supplementary Table 9: Intracellular cytokine functions in monocytes** | | | |
| --- | --- | --- | --- |
| **Cytokine**  **markers** | **Role in**  **Inflammation** | **Marker function** | **Reference** |
| IL-10 | Anti-inflammatory | Induces regulatory role in neuroinflammatory diseases | Chuluundorj, D., et al. 2014 |
| TGF-β |  |  | Di Pardo, A., et al. 2013 |
| GM-CSF | Inflammatory | Promotes the migration of monocytes to the CNS | Vogel, D.Y., et al., 2015 |
| IL-6 | Pro and  anti-inflammatory | Induces a protective  role during  neuroinflammation | Fiedler, S.E., et al., 2017 |
| TNF-a | Pro-inflammatory | mediates several chronic inflammatory diseases | Fiedler, S.E., et al., 2017 |

**Supplementary Table 10: scRNA-seq distribution number of “CNS-homing” and “Non-CNS-homing” monocytes in patients with PANS (flare and recovery) and healthy control**

| Samples | "CNS-homing" monocytes | "Non-CNS-homing" monocytes |
| --- | --- | --- |
| PANS Flare 1 | 476 | 98 |
| PANS Flare 2 | 178 | 43 |
| PANS Flare 3 | 466 | 95 |
| PANS Flare 4 | 391 | 77 |
| PANS recovery 1 | 417 | 126 |
| PANS recovery 2 | 1168 | 368 |
| PANS recovery 3 | 879 | 241 |
| PANS recovery 4 | 606 | 214 |
| Healthy Control 1 | 54 | 81 |
| Healthy control 2 | 209 | 241 |
| Healthy control 3 | 411 | 441 |

| **Supplementary Table 11: Surface markers tested and their functions in monocytes and monocyte-derived cells** | | | | |
| --- | --- | --- | --- | --- |
| **Cell type** | **Role in inflammation** | **Surface markers** | **Marker function** | **Reference** |
| Classical monocyte  (M1-polarized monocyte)  CD14+ | Pro-inflammatory | CD64 | involved in macrophage antibody-dependent cellular cytotoxicity and clearance of immune complexes | Atri, C. et. al. 2018 |
|  |  | CD86 | provides costimulatory signals for T cell activation |  |
| Classical  Monocyte(M2-polarized) CD14+ | Anti-inflammatory | CD163 | innate immune sensor for gram+ and gram- bacteria |  |
|  |  | CD206 | endocytosis phagocytosis, and scavenging of mannoglycoproteins |  |
| Monocyte-derived dendritic cell | Pro-inflammatory | CD11b | integrin receptor involved in immune cell adhesion, migration, and phagocytosis | Segura, E. and S. Amigorena 2013 |
|  |  | CD11c | defining marker for DCs; involved in phagocytosis | Tang-Huau, T.-L. and E. Segura 2019 |
|  |  | CD209 | cell adhesion molecule and pathogen receptor |  |
| Convential/Myeloid  Dendritic Cell  (cDC1) | Antigen cross-presentation | CD141 | Endothelial cell receptor for thrombin | Minoda Y. et. al. 2017 |
| “CNS-homing’’monocyte CD14+ | Unknown | CCR2 | Receptor for the CCL2 chemokine | Wohleb, E.S., et al. 2013 |
|  |  | CX3CR1 | Receptor for the fractalkine (CX3CL1) chemokine |  |
|  |  | VLA-4 | Integrin dimer (CD49d/CD29); primary ligands include VCAM-1. | Takeshita, Y. and R.M. Ransohoff 2012 |
|  |  | CD166 | Adhesion molecule, Ig superfamily member; mediates homophilic interactions with brain endothelial cells. | Lyck, R., et al. 2017 |

**
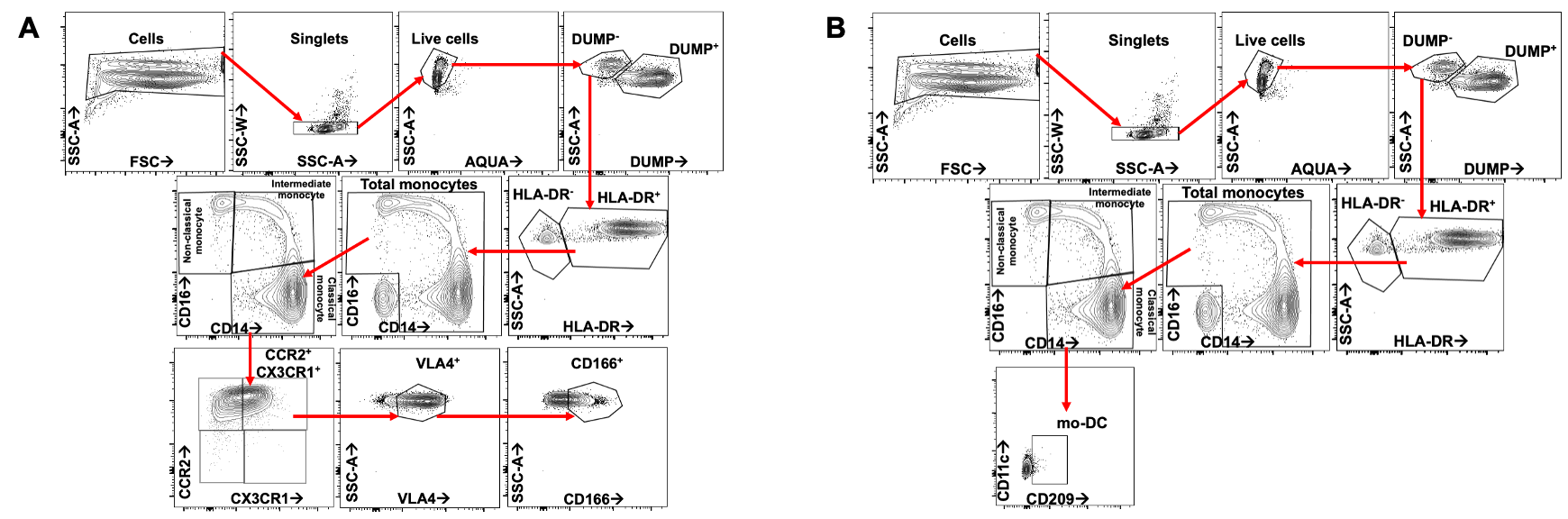
**

**
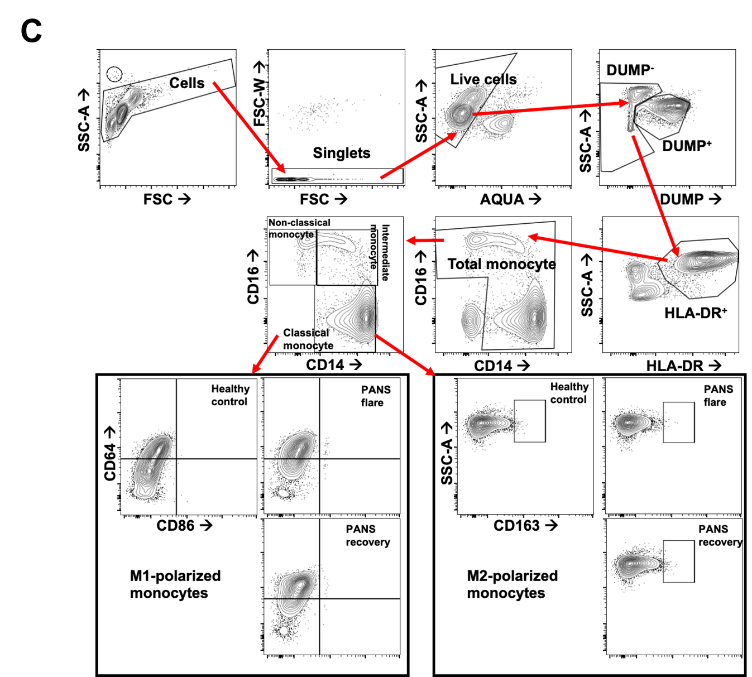
**

**Figure S1. Gating strategies to identify “CNS-homing” monocytes, monocyte-derived dendritic cells, M1 and M2- Polarized monocytes.** Unstimulated PBMCs were stained with fluorophore-tagged antibodies to identify **(A)** subsets of monocytes and **(B)** monocyte-derived dendritic cells**(C)** M1 and M2- polarized monocytes. Representative contour-plot shows the gating strategy used to identify each subset of monocytes and dendritic cells.


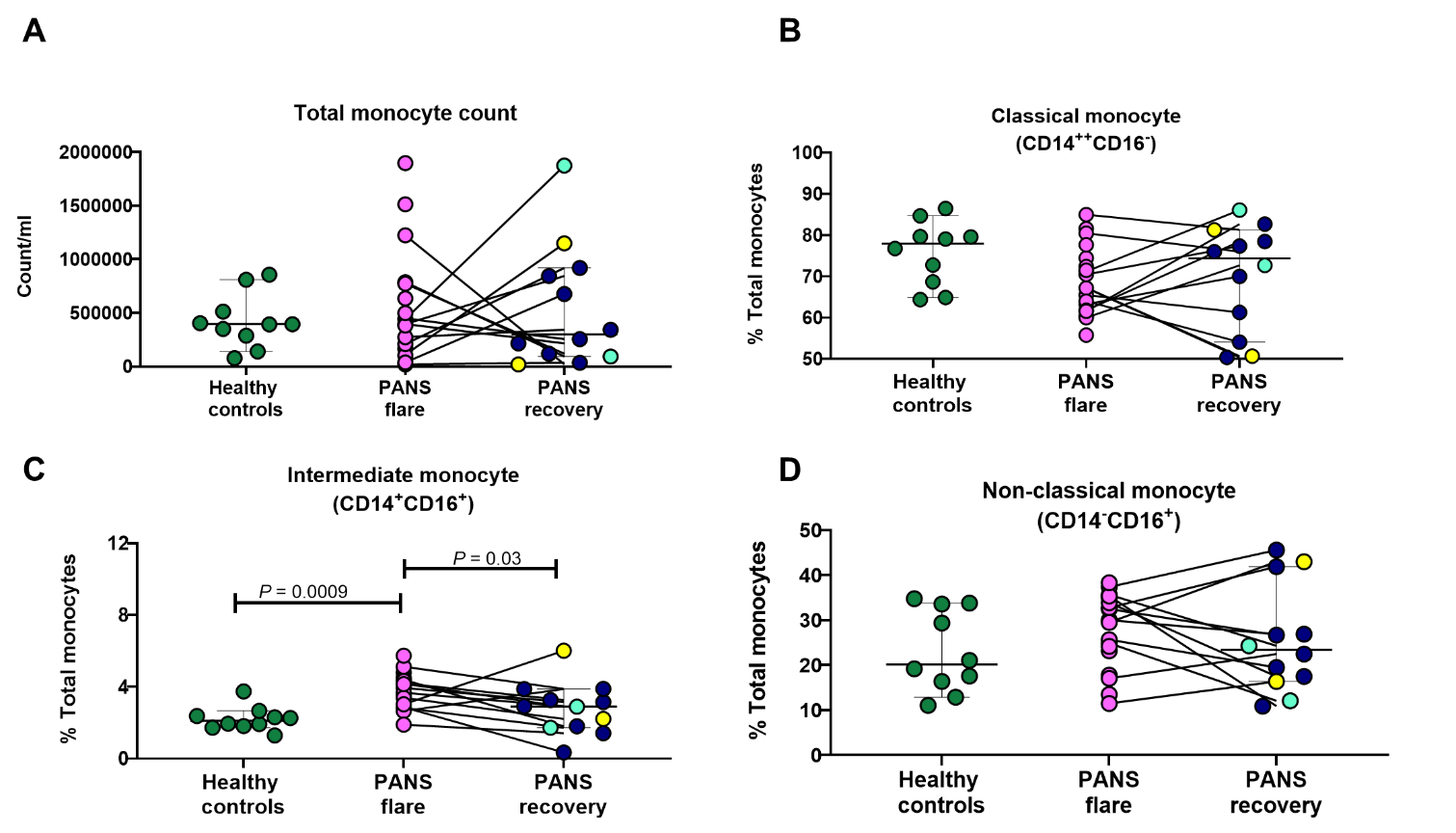


**Figure S2. Absence of pronounced monocytosis in female patients with PANS.**

Unstimulated PBMCs from female patients with PANS in flare (n=12 ) and recovery (partial or full) (n=12 )(paired samples, n=12 ) and age/sex/race/ethnicity- matched healthy controls (n=10) were stained with markers for monocyte subsets. **(A)** Total monocytes count in female subjects. **(B, C, D)** Proportion of classical, intermediate, and non-classical monocyte in female subjects (respectively), represented as % of total monocyte. HLA-DR^+^ population was manually gated to identify total monocytes and the 3 major monocyte subsets based on the surface expression of CD14^+^ and CD16^+^: classical (CD14^+^), non-classical (CD16^+^) and intermediate (CD14^+^CD16^+^). CD14^+^ cells were further gated to identify monocyte-derived dendritic cell (mo-DC; see supplementary Figure S1). Paired samples are indicated by a straight line and were analyzed by Friedman Test (P value < 0.05 indicating significance). Unpaired samples were analyzed by non-parametric Mann-Whitney test (P value < 0.05 indicating significance). The different colors displayed in the recovery groups represent three sub-groups of patients whose state and course (prior to their recovery) were characterized by: new-onset (light blue), flare-on-persistent baseline (dark blue) and static-persistent (yellow).


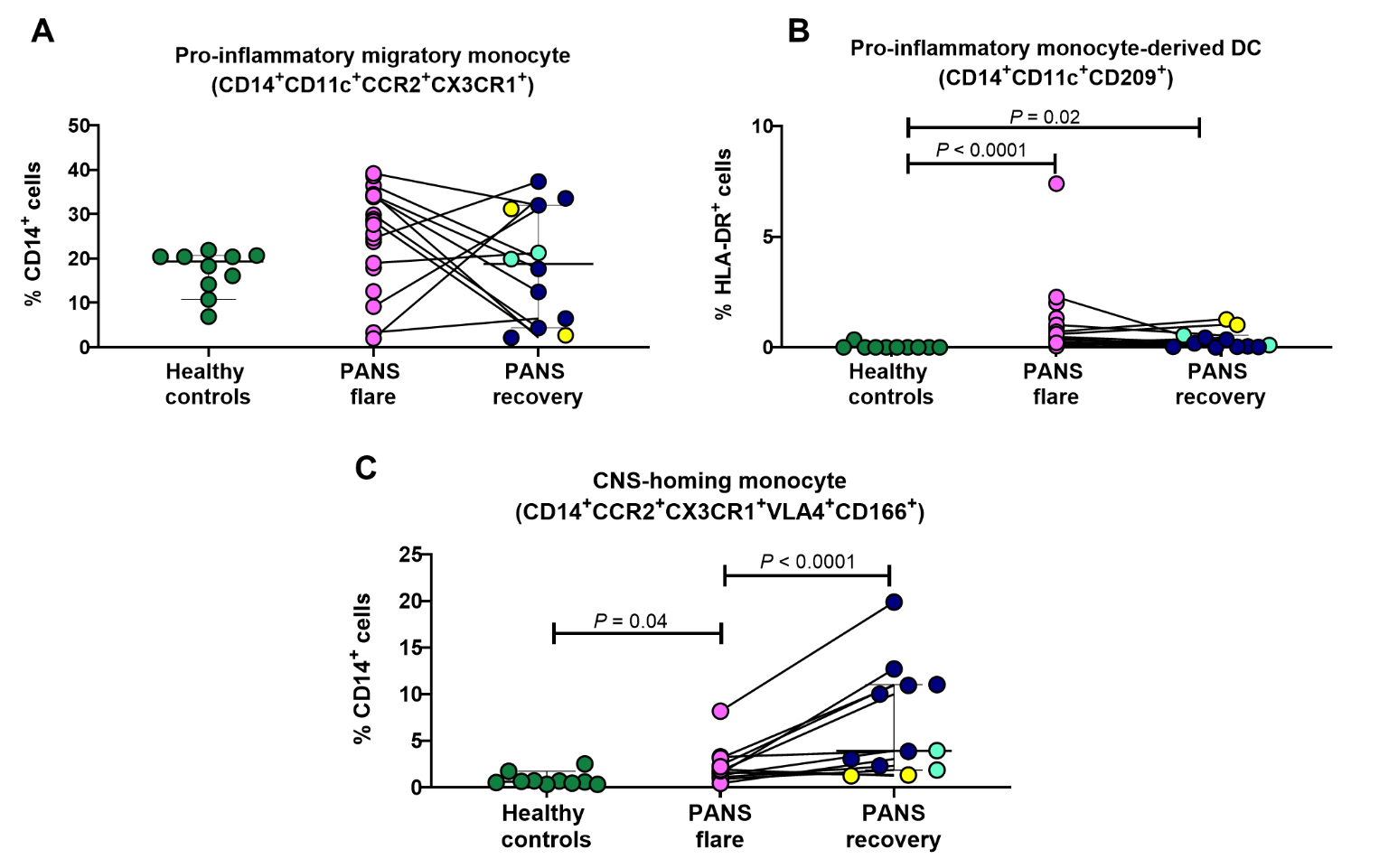


**Figure S3. Status of pro-inflammatory migratory monocytes, monocyte-derived dendritic cells, and “CNS-homing” monocytes in female patients with PANS.** Unstimulated PBMCs from patients with PANS in flare (n=12 ) and in recovery (partial or full) (n= 12)(paired samples, n=12 ) and age/ (female)/ethnicity-matched controls (n=10) were stained with marker panels defining migratory monocytes, monocyte-derived dendritic cells, and “CNS-homing” monocytes. **(A)** Proportion of pro-inflammatory migratory monocytes (CD14^+^CD11c^+^CR2^+^CX3CR1^+^) are presented as a percent of CD14^+^ cells. The gates were set based on the fluorescence minus one (FMO) plot of CD11c, CCR2, and CX3CR1. **(B)** Monocyte-derived dendritic cells were gated and identified within HLA-DR^+^ cells as CD14^+^CD11b^+^CD11c^+^CD209^+^ population. CD209 gates were set based on the absence of CD209^+^ population within the CD16^+^ population; CD209 is not expressed by non-classical monocytes (Cai et al., 2021). **(C)** Frequency of “CNS-homing” monocytes (CD14^+^CCR2^+^CX3CR1^+^VLA4^+^CD166^+^) are presented as a percent of CD14^+^ cells. Paired samples are indicated by a straight line and were analyzed by Friedman Test (*P* value < 0.05 indicating significance). Unpaired samples were analyzed by non-parametric Mann-Whitney test (*P* value < 0.05 indicating significance). The different colors displayed in the recovery groups represent three sub-groups of patients whose state and course (prior to their recovery) were characterized by: new-onset (light blue), flare-on-persistent baseline (dark blue) and static-persistent (yellow).


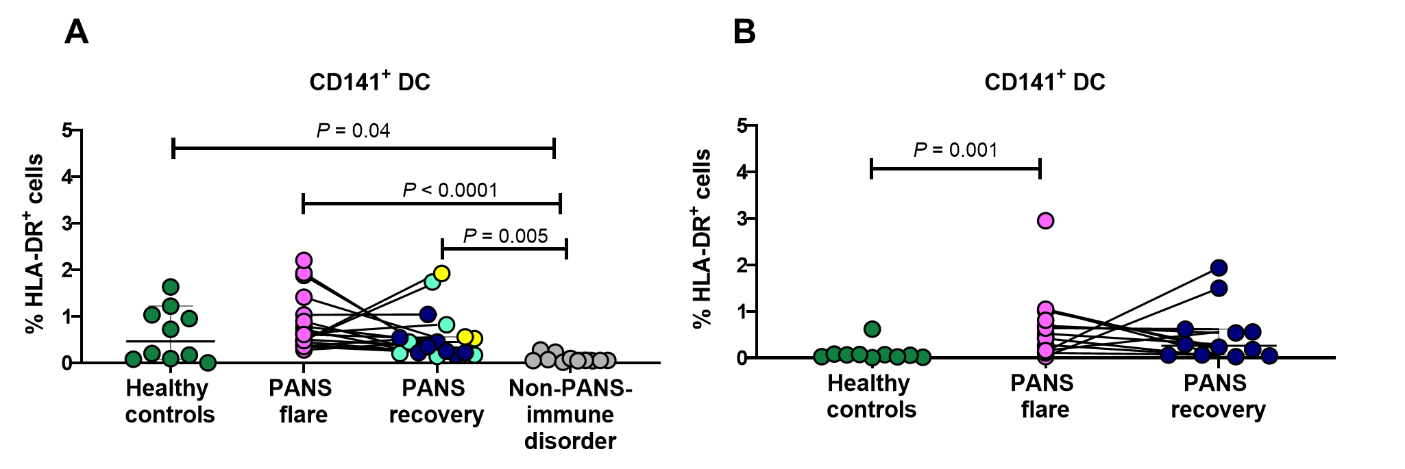


**Figure S4. Conventional/myeloid dendritic cell subset (cDC1)(expressing CD11c and CD141) in male and female patients with PANS.** Unstimulated PBMCs from patients with PANS in flare (n=12 ) and in recovery (partial or full) (n=12 )(paired samples n=12 ), age/sex/race/ethnicity matched healthy controls (n=10 ) and non-PANS immune disorder (patients with brain diseases and asthma) were stained with marker panels defining conventional DC1 (cDC1). Proportion of CD14^-^CD16^-^CD11c^+^ HLA-DR^+^ CD141^+^ cells are presented as a percent of HLA-DR^+^ cells in **(A)** male subjects, and **(B)** female subjects. The gates were set based on the fluorescence minus one (FMO) plot of CD11c, CD141. Paired samples are indicated by a straight line and were analyzed by Friedman Test (*P* value < 0.05 indicating significance). Unpaired samples were analyzed by non-parametric Mann-Whitney test (*P* value < 0.05 indicating significance). The different colors displayed in the recovery groups represent three sub-groups of patients whose state and course (prior to their recovery) were characterized by: new-onset (light blue), flare-on-persistent baseline (dark blue) and static-persistent (yellow).


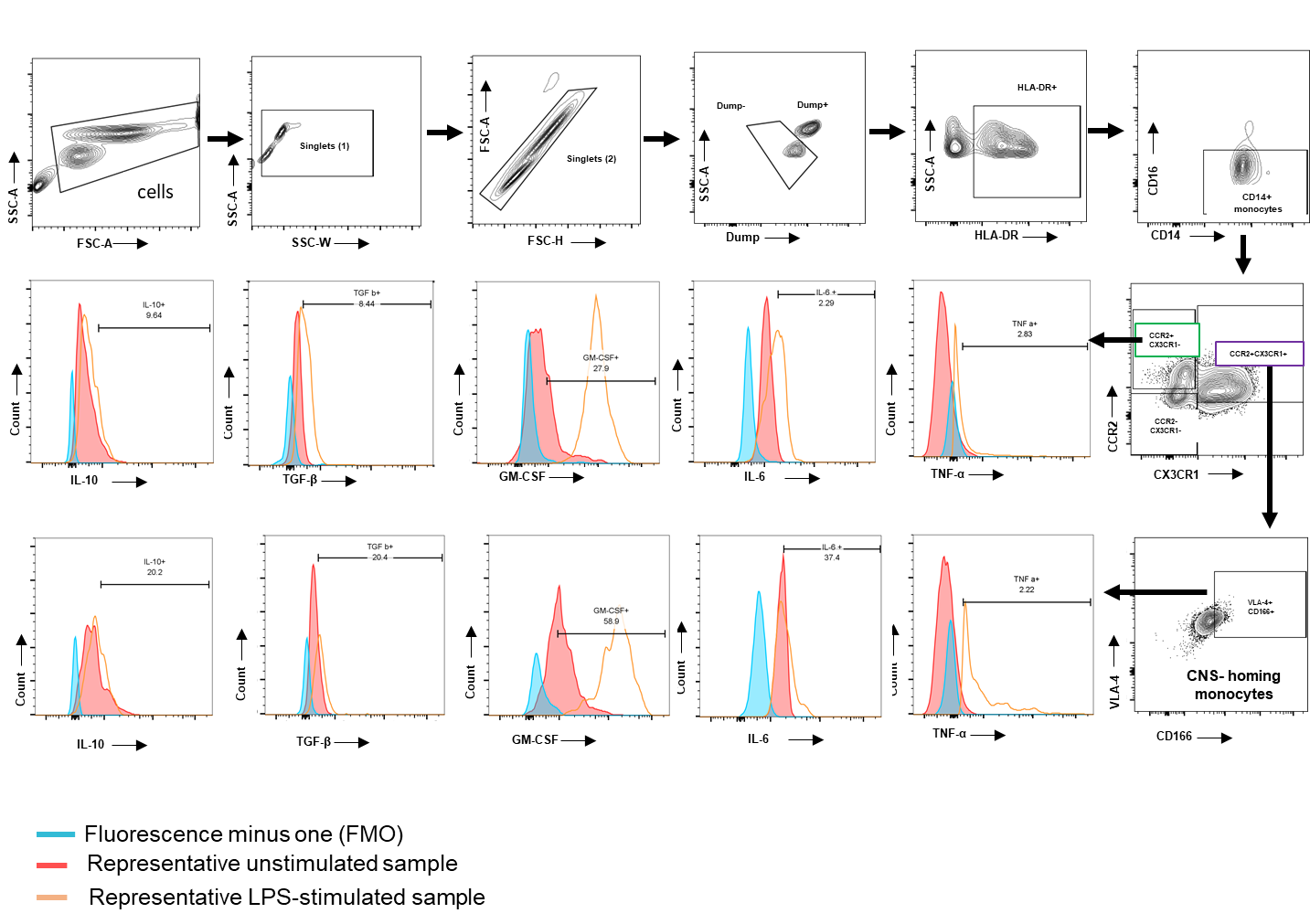


**FigureS5. Gating strategies of intracellular cytokines in “CNS-homing”**  **monocytes** (**CD14^+^CCR2^+^CX3CR1^+^VLA4^+^CD166^+^** ) **vs “non-CNS-homing”** **monocytes (CD14+CCR2+CX3CR1-).** Unstimulated- smart tubes fixed whole blood samples were stained with fluorophore-tagged antibodies to measure the intracellular cytokines production in monocytes subsets. Extracellular dump channel markers (CD235a,CD15,CD3,CD19,CD56, CD66b) were used to exclude erythrocytes, granulocytes T cells, B cells, NK cells, and neutrophils, respectively from the analysis. Representative histograms used to identify each cytokine. The gates were set based on the fluorescence minus one (FMO) and 1µg/ml LPS- stimulated plot of IL-10, TGF-β, GM-CSF, IL-6, and TNF-α in “CNS-homing” and “non-CNS-homing” monocytes.

**
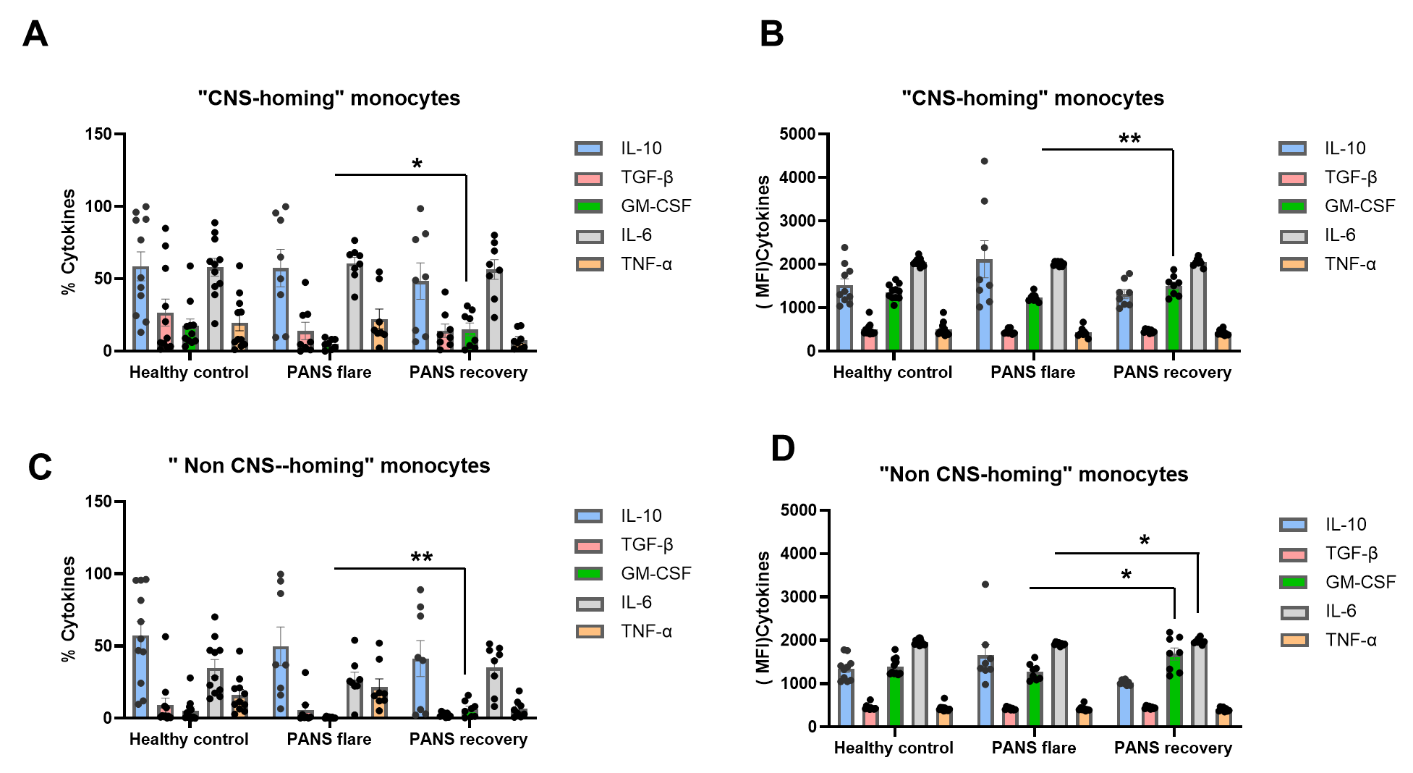
**

**FigureS6.** The percentages (A, C) and median fluorescence intensities (MFI) (B, and D) of cytokine-producing cells in “CNS-homing” and “ non-CNS-homing” monocytes across all clinical states—PANS flare, PANS recovery , and healthy controls. p<0.05, **p<0.01, ***p<0.001,****p<0.0001; significant differences were determined using the, nonparametric Wilcoxon’s test to compare the paired samples PANS flare vs PANS recovery and the nonparametric Mann-Whitney test to compare healthy control vs PANS flare and PANS recovery.

**
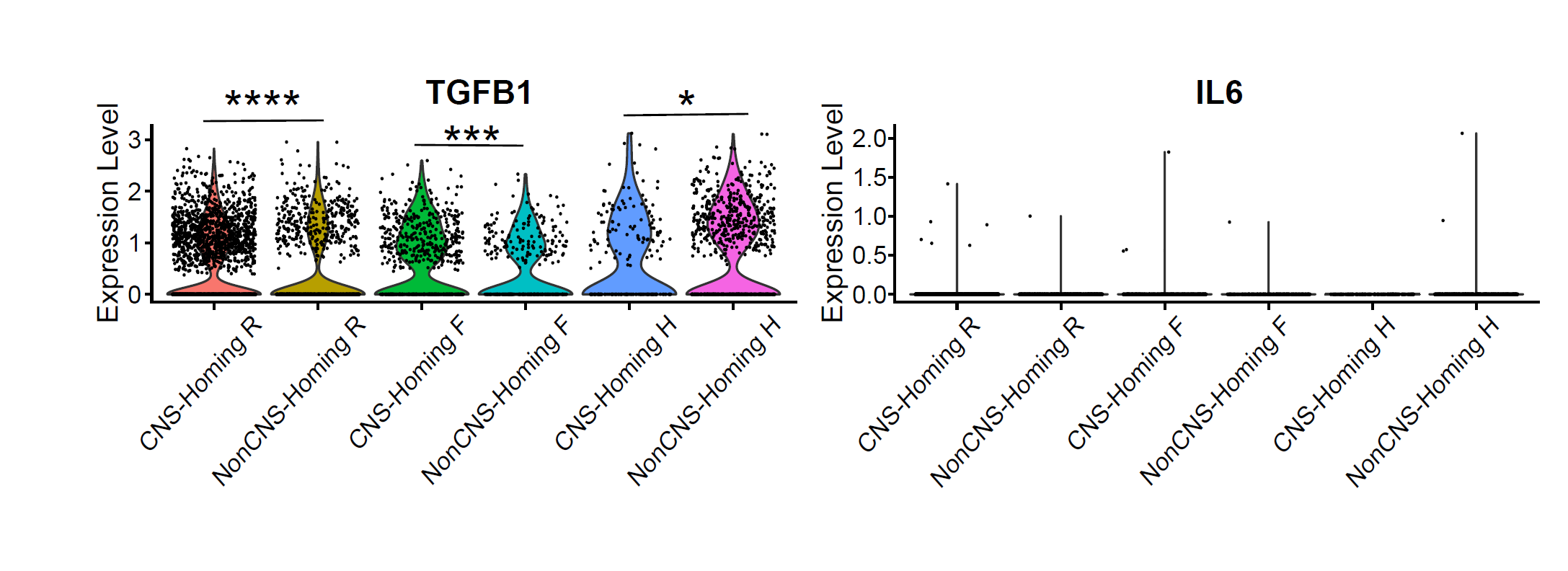

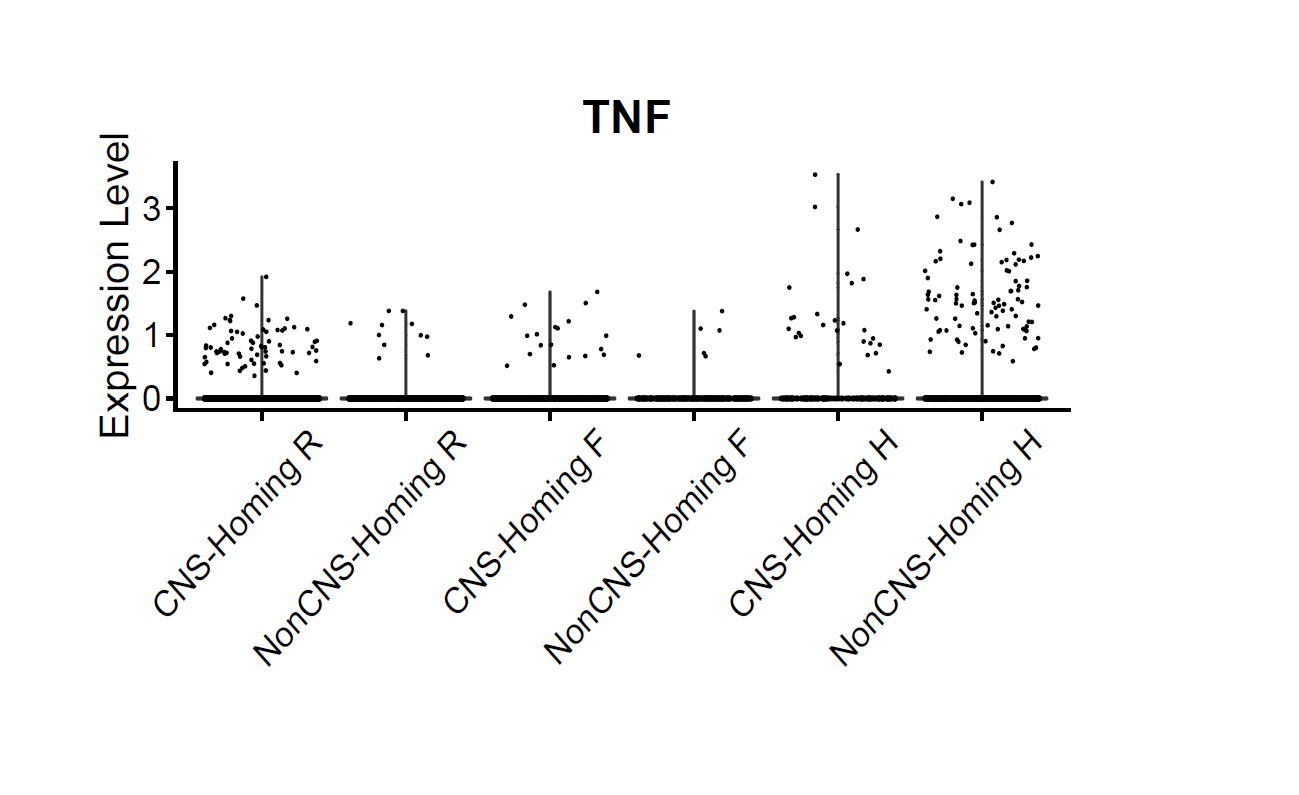
**

**FigureS7.Expressions of cytokines genes in “CNS-homing” vs “non-CNS-homing” monocytes in PANS.** Violin plots are used to show the expression levels of TGF-(TGFB1), IL6 and TNF-(TNF) genes in the “CNS-homing” vs “non-CNS-homing” monocytes cell types identified by single-cell transcriptomics in PANS(flare(F), recovery (R)), healthy control(H). Each dot represents a single cell. The x-axis identifies the cell types; “CNS-homing” and “non-CNS-homing” .The y-axis represents an expression of genes in log (TPM). n=4. The *p*-value evaluation on the violin plot are performed using Wilcoxon rank-sum test for pairwise comparisons (*=*p-value*<0.05, **=*p-value* <0.01, ***=*p-value* <0.001,**** =*p-value* <0.0001").

**References**

1. Masterson, E.E., et al., *Defining clinical course of patients evaluated for pediatric acute-onset neuropsychiatric syndrome (PANS): phenotypic classification based on 10 years of clinical data.* Dev Neurosci, 2025: p. 1-33.

2. Chain, J.L., et al., *Autoantibody Biomarkers for Basal Ganglia Encephalitis in Sydenham Chorea and Pediatric Autoimmune Neuropsychiatric Disorder Associated With Streptococcal Infections.* Front Psychiatry, 2020. **11**: p. 564.
